# Subgenomic Stability of Progenitor Genomes During Repeated Allotetraploid Origins of the Same Grass *Brachypodium hybridum*

**DOI:** 10.1093/molbev/msad259

**Published:** 2023-11-24

**Authors:** Wenjie Mu, Kexin Li, Yongzhi Yang, Adina Breiman, Jiao Yang, Ying Wu, Mingjia Zhu, Shuai Wang, Pilar Catalan, Eviatar Nevo, Jianquan Liu

**Affiliations:** State Key Laboratory of Herbage Innovation and Grassland Agro-Ecosystem, College of Ecology, Lanzhou University, Lanzhou 730000, China; State Key Laboratory for Animal Disease Control and Prevention, College of Veterinary Medicine, Lanzhou University, Lanzhou Veterinary Research Institute, Chinese Academy of Agricultural Sciences, Lanzhou, China; State Key Laboratory of Herbage Innovation and Grassland Agro-Ecosystem, College of Ecology, Lanzhou University, Lanzhou 730000, China; State Key Laboratory of Herbage Innovation and Grassland Agro-Ecosystem, College of Ecology, Lanzhou University, Lanzhou 730000, China; Department of Evolutionary and Environmental Biology, University of Tel-Aviv, Tel-Aviv 6997801, Israel; State Key Laboratory of Herbage Innovation and Grassland Agro-Ecosystem, College of Ecology, Lanzhou University, Lanzhou 730000, China; State Key Laboratory of Herbage Innovation and Grassland Agro-Ecosystem, College of Ecology, Lanzhou University, Lanzhou 730000, China; State Key Laboratory of Herbage Innovation and Grassland Agro-Ecosystem, College of Ecology, Lanzhou University, Lanzhou 730000, China; State Key Laboratory for Animal Disease Control and Prevention, College of Veterinary Medicine, Lanzhou University, Lanzhou Veterinary Research Institute, Chinese Academy of Agricultural Sciences, Lanzhou, China; Escuela Politecnica Superior de Huesca, Universidad de Zaragoza, Huesca 22071, Spain; Institute of Evolution, University of Haifa, Haifa 3498838, Israel; State Key Laboratory of Herbage Innovation and Grassland Agro-Ecosystem, College of Ecology, Lanzhou University, Lanzhou 730000, China

**Keywords:** allopolyploids, comparative genomics, drought response, ecological adaptation, homeologous exchanges, homeologous expression bias, subgenome dominance, recurrent origins

## Abstract

Both homeologous exchanges and homeologous expression bias are generally found in most allopolyploid species. Whether homeologous exchanges and homeologous expression bias differ between repeated allopolyploid speciation events from the same progenitor species remains unknown. Here, we detected a third independent and recent allotetraploid origin for the model grass *Brachypodium hybridum*. Our homeologous exchange with replacement analyses indicated the absence of significant homeologous exchanges in any of the three types of wild allotetraploids, supporting the integrity of their progenitor subgenomes and the immediate creation of the amphidiploids. Further homeologous expression bias tests did not uncover significant subgenomic dominance in different tissues and conditions of the allotetraploids. This suggests a balanced expression of homeologs under similar or dissimilar ecological conditions in their natural habitats. We observed that the density of transposons around genes was not associated with the initial establishment of subgenome dominance; rather, this feature is inherited from the progenitor genome. We found that drought response genes were highly induced in the two subgenomes, likely contributing to the local adaptation of this species to arid habitats in the third allotetraploid event. These findings provide evidence for the consistency of subgenomic stability of parental genomes across multiple allopolyploidization events that led to the same species at different periods. Our study emphasizes the importance of selecting closely related progenitor species genomes to accurately assess homeologous exchange with replacement in allopolyploids, thereby avoiding the detection of false homeologous exchanges when using less related progenitor species genomes.

## Introduction

Allopolyploidy plays a significant role in driving plant speciation and has had a profound impact on the genomes and phenomes of most angiosperms (Soltis et al. 2009; Van de Peer et al. 2017). All modern diploid flowering plants are considered downsized paleopolyploids, as they have returned to the diploid state by eliminating redundant sequences (Doyle et al. 2008; Jiao et al. 2011; Soltis et al. 2016). Some small genome diploids, like *Brachypodium*, have experienced several rounds of polyploidization and diploidization throughout their history (Salse et al. 2008). Recent meso- and neopolyploids in *Brachypodium* and other grasses have emerged through new hybridizations and whole genome duplication (WGD) events (Stebbins 1985; Soltis and Soltis 2021; Sancho et al. 2022), a process which is still ongoing today. Allopolyploid speciation appears to occur rapidly due to the effective reproductive isolation of the allopolyploid from its progenitor species (Te Beest et al. 2012; Oxelman et al. 2017). In fact, biological competition, niche competition, or better adaptation to new environments could lead to the rapid displacement of parental populations by successful hybrid allopolyploids (Leitch and Leitch 2008). Polyploidy is widely recognized as a significant source of genomic and evolutionary novelty, contributing to adaptive speciation (Soltis et al. 2014; Van de Peer et al. 2017; Baduel et al. 2018; Nieto Feliner et al. 2020). However, to truly understand the impact of polyploidy on evolutionary innovation and the formation of new species, it is essential to analyze it within a comprehensive pangenomic framework that takes into account both the progenitor species and the resulting allopolyploids. This analysis assists in revealing the genomic diversity of allopolyploid species, which is a combination of the diversities inherited from their progenitor genomes and those acquired through hybridization, polyploidization, and diploidization with evolutionary novelty (Gordon et al. 2020; Scarlett et al. 2022).

One of the immediate purported consequences attributed to allopolyploidy is the need for genomic and regulatory changes to accommodate the combination of different genomes within a single nucleus (Edger et al. 2019). These alterations aim to stabilize the mixed nuclear environment, and as a result, homeologous exchanges (HEs), which involve chromosomal recombinations between homeologs, have been observed in both recently synthesized and natural plant allopolyploids (e.g. *Tragopogon*, *Gossypium*, *Brassica*, *Fragaria*, *Cucumis*) (Chester et al. 2012; Chalhoub et al. 2014; Guo et al. 2014; Edger et al. 2019; Yu et al. 2021; Deb et al. 2023). HEs are believed to be responsible for generating evolutionary novelty, phenotypes, and contributing to speciation (Mason and Wendel 2020; Deb et al. 2023). However, other synthetic or natural allopolyploids, including *Arabidopsis*, *Capsella*, *Trifolium*, and *Eragrostis*, do not exhibit evidence of homeologous recombinations (Douglas et al. 2015; Griffiths et al. 2019; VanBuren et al. 2020; Burns et al. 2021). Another consequence often associated with allopolyploidy is subgenome dominance or homeolog expression bias (HEB), which is considered a mechanism that facilitates genome stabilization by resolving (epi)genetic conflicts in the allopolyploid (Edger et al. 2019). Subgenome dominance correlates with HEs when HE involves replacement (deletion/duplication) (Deb et al. 2023). When the dominant subgenome contains a greater number of gene copies and highly expressed homeologs compared to the submissive subgenome, it also leads to subgenome dominance (Alger and Edger 2020). Subgenome dominance can be established immediately after WGD or gradually over evolutionary time (Flagel and Wendel 2010; Edger et al. 2017). It is believed that subgenome dominance has contributed to the adaptive success of the diploidized allopolyploid, with a number of evidences from different angiosperm groups (Alger and Edger 2020; Deb et al. 2023). However, some allopolyploids do not exhibit significant HEB (Scarlett et al. 2022), which contradicts the expected pattern. The recently proposed “genome chimera” model offers a new perspective to reconcile these conflicting patterns within a comprehensive framework. It suggests that subgenome dominance, particularly HEB, simply reflects the differences in genomic background in terms of gene expression and regulation (e.g. transcription efficiency) between the two progenitor genomes, rather than being an inherent evolutionary trait of neo-allotetraploids (Zhang et al. 2023).

Meiosis-regulating genes play a crucial role in the cytogenetic mechanisms that influence plant response to hybridization and WGD, as they are responsible for ensuring homologous pairing and stabilizing nascent allopolyploids (Jenczewski and Alix 2004). In *Triticum* and *Brassica* allopolyploids, candidate meiotic genes that prevent meiotic crossovers between homeologs have been identified, thereby avoiding mis-segregation, aneuploidy, and gamete infertility (Martín et al. 2018; Higgins et al. 2021). Notably, *Ph1* and *Ph2*-type genes have shown major qualitative effects, particularly in allopolyploid wheat (Martín et al. 2021; Serra et al. 2021). The establishment of meiotic stability after allopolyploidization has occurred gradually over generations in certain angiosperm groups, possibly due to selection for particular allele combinations that confer higher fertility (Cifuentes et al. 2010; Mason and Wendel 2020), while in other recent polyploids meiotic stability might have been acquired immediately (Jenczewski et al. 2002). The rapid stability of allopolyploids may depend on the divergence of progenitors (Ramsey and Schemske 2002), progenitor genotypes (Steige and Slotte 2016), and the epigenetic effects of transposable elements (TEs; Vicient and Casacuberta 2017; Wendel et al. 2018). Although the structural and functional impact of subgenomes on the adaptive success of new allopolyploid phenotypes in specific ecological niches has been investigated in several plant polyploids (van de Peer et al. 2021), it has not been fully assessed within a multiple-origin evolutionary scenario of the same polyploid species.

*Brachypodium hybridum* has emerged as a model system for stable allopolyploidy in grasses, specifically immediate amphidiploidy after WGD (Catalán et al. 2012; Scholthof et al. 2018; Gordon et al. 2020; Hasterok et al. 2022; Scarlett et al. 2022). Previous phylogenetic and cytogenetic studies have demonstrated that *B. hybridum* (2*n* = 4*x* = 30, *x* = 10 + 5), an allotetraploid grass, originated recurrently and bidirectionally from two extant diploid progenitor species *B. stacei* (2*n* = 2*x* = 20, *x* = 10) and *B. distachyon* (2*n* = 2*x* = 10, *x* = 5) (Catalán et al. 2012; Lopez-Alvarez et al. 2012; Scholthof et al. 2018). The native Mediterranean niche of *B. hybridum* overlaps with those of its progenitor species, with higher overlap observed with the more mesic *B. distachyon* niche in the northern part of the Mediterranean range and with the more aridic *B. stacei* niche in the southern part of the range (López-Alvarez et al. 2015). Genome-wide analyses of *B. hybridum* and its progenitor species have dated the recurrent origins of the allotetraploid to two different geological times in the Western Mediterranean region, approximately 1.4 million years ago (Ma) for the ancestral D-plastotypes (D-anc) (maternal parent *B. distachyon*) and 0.14 Ma for the recent S-plastotypes (Western-Med) (maternal parent *B. stacei*) (Gordon et al. 2020; Hasterok et al. 2022; Scarlett et al. 2022). *B. hybridum* also served as control for testing the accuracy of phylogenetic subgenomic detection algorithms, as it is the only polyploid species of the genus with known extant diploid progenitor genomes while all the others have one or more “ghost” subgenomes (Sancho et al. 2022). Both allopolyploid hybrids have displayed high subgenomic integrity with respect to the progenitor species genomes, although the D-anc has shown evidence of post-WGD structural change and slight but significant biased subgenomic gene loss (Scarlett et al. 2022). However, it is important to note that these results were based on comparative genomics and phylogenomics using incomplete Illumina-based reference genomes of *B. hybridum* S-recent plastotype (West-Med), *B. stacei*, and *B. distachyon*, a more complete PacBio-based reference genome of *B. hybridum* D-anc plastotype, and low resequenced genomes of several other *B. hybridum* and *B. distachyon* accessions (Gordon et al. 2020; Scarlett et al. 2022). Additionally, analyzing the *B. hybridum* D-anc genome presents a challenge due to the considerable divergence of its BhS and BhD subgenomes compared to their respective *B. stacei* and *B. distachyon* progenitor lineages, as the two subgenomic lineages split well before the recent radiations of the current *B. stacei* and *B. distachyon* clades (Gordon et al. 2020).

Here, we conducted a comprehensive analysis of this allopolyploid model system by employing comparative genomics and evolutionary analyses. We extensively studied a wide range of *B. hybridum* accessions from the relatively unexplored Eastern Mediterranean region, as well as other Mediterranean localities. Our research involved the generation of a complete PacBio-based reference genome for an accession from the East-Med region, along with several population-level resequenced genomes. To ensure accurate assessments, we selected the most closely related progenitor genomes from the *B. stacei* (Gordon et al. 2020; Mu et al. 2023) and *B. distachyon* (Gordon et al. 2017) pangenomes. Our analysis revealed a novel origin for a *B. hybridum* S-plastotype in the Eastern Mediterranean (East-Med) region, specifically in Evolution Canyon I (ECI), and other localities of Israel. These populations emerged from distinct local ancestors and are distributed within a more arid latitudinal belt compared to the previously known ancestral and recent *B. hybridum* populations in the west (supplementary fig. S1 and supplementary table S1, Supplementary Material online). ECI provides an ideal geographic setting for investigating microscale evolutionary models, as it encompasses two contrasting slopes: the xeric African south-facing slope with ecotypes adapted to drought and heat stresses and the forested European north-facing slope with ecotypes adapted to more mesic habitats and less stressful conditions (Nevo 2012). Utilizing the *B. hybridum* grass complex as a model system, we examined the extent of HEs, HEB, and the meiotic regulation mechanisms that contribute to the rapid stability of the allotetraploids within a time-course evolutionary scenario involving three recurrent origins of *B. hybridum* in different geographical and ecological contexts. Furthermore, we uncovered the functional contributions of the diploid progenitor subgenomes to the adaptive success of the newly discovered *B. hybridum* allotetraploid plants to the arid conditions of the African slope (AS) in ECI, Israel.

## Results

### Genomic Landscape of *Brachypodium hybridum* ECI

Combining PacBio HiFi and Hi-C sequencing technologies, we generated the de novo assembled *Brachypodium hybridum* ECI (Bhyb-ECI) reference genome (supplementary fig. S2 and supplementary table S2, Supplementary Material online). The final genome assembly captured 527.87 Mb base pairs with 15 pseudo-chromosomes (supplementary fig. S2B and supplementary tables S2A and B, Supplementary Material online). The genome size of Bhyb-ECI is similar to those of the reference *B. hybridum* S-plastotype (Bhyb-ABR113) genome (530.5 Mb) and the reference *B. hybridum* D-plastotype (Bhyb-26) genome (528.49 Mb) (Joint Genome Institute [JGI]; Phytozome, https://genome.jgi.doe.gov/). The de novo assembly of the Bhyb-ECI reference genome showed a high contiguity, the contig N50 was 18.70 Mb, and most chromosomes were composed of fewer than five contigs and with few gaps (supplementary fig. S2C and supplementary table S2D, Supplementary Material online). The base call accuracy (QualityValue [QV]) and assembly completeness were 38.79% and 99.38% with >99% and >98.5% mapping and coverage rates according to Illumina paired-end read mapping, respectively (supplementary table S2E, Supplementary Material online). Almost complete sets of Benchmarking sets of Universal Single-Copy Orthologs (BUSCO) genes (98.8%) were detected in the Bhyb-ECI assembly (supplementary fig. S2D, Supplementary Material online). In addition, the LTR Assembly Index (LAI) indicated a high long-terminal repeat (LTR) retrotransposon completeness (supplementary fig. S2C, Supplementary Material online), showing values higher than those reported for Bhyb-ABR113 (v1.1), but similar to those of Bhyb-26. Together, these metrics demonstrate high consistency and completeness of the new genome assembly, as well as greatly improved contiguity and repetitive sequence completeness.

We predicted 72,685 high-confidence protein coding genes (supplementary table S3A, Supplementary Material online). The average coding sequence region length, exon length, and exon number are similar to those of other representative genomes of the *B. hybridum*, *B. stacei*, and *B. distachyon* species (supplementary fig. S3A, Supplementary Material online). More than 96% of the predicted genes of the Bhyb-ECI genome had homologs in public functional databases (supplementary table S3B, Supplementary Material online). BUSCO analysis in the Bhyb-ECI genome indicated that 99.8% complete genes were present in our gene model predictions (supplementary fig. S2D, Supplementary Material online), indicating the high completeness of the gene model annotation. Most BUSCO genes (98.8%) were retained in duplicate, corresponding to homeologs present in both subgenomes of the allotetraploid. A total of 3,764 genes in the Bhyb-ECI genome were classified as putative transcription factors, belonging to 67 gene families and representing 5.1% of the total predicted genes (supplementary table S3C, Supplementary Material online). Moreover, 219.62 Mb of repetitive element sequences accounted for 41.60% of the total Bhyb-ECI genome (supplementary table S3D, Supplementary Material online); these figures were similar to those detected in previous *B. hybridum* assemblies (Bhyb-ABR113, 38.13%, Gordon et al. 2020; Bhyb-26, 41.51%, Scarlett et al. 2022).

**Fig. 1.**
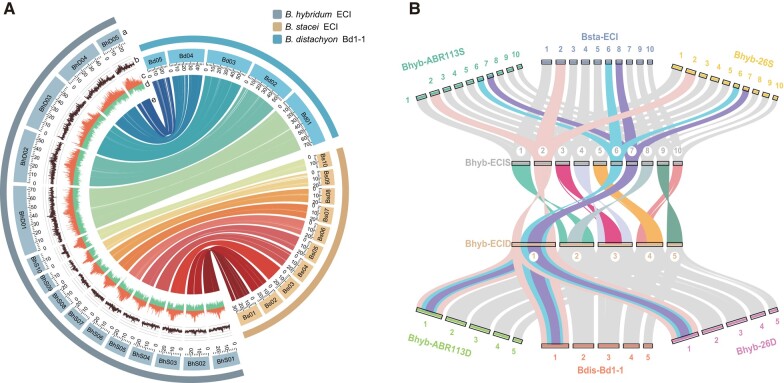
Genome organization and evolutionary landscape of the newly sequenced *Brachypodium hybridum* Bhyb-ECI reference genome. A) Overview of *B. hybridum* Bhyb-ECI genome assembly/synteny with progenitor species genomes *B. stacei* Bsta-ECI and *B. distachyon* Bdis-Bd1-1. The tracks indicate: (a) chromosomes, (b) GC contents, (c) TE densities, (d) gene models' densities, and (e) collinearity of syntenic genes. B) Conserved synteny between the two progenitor species' genomes and the respective *B. hybridum* (Bhyb-ECI, Bhyb-ABR113, and Bhyb-26) BhD and BhS subgenomes showing highly structural divergence of chromosomes from the two subgenomes, consistent with the high divergence between chromosomes of the two corresponding progenitors' genomes.

We partitioned the Bhyb-ECI genome into two subgenomes based on the collinearity of its sequence to the genomes of the respective progenitor species (Fig. 1). The *B. distachyon-*like subgenome (BhD) consisted of 5 chromosomes and 38,330 genes (275.83 Mb), while the *B. stacei-*like subgenome (BhS) comprised 10 chromosomes and 33,766 genes (252.04 Mb) (Fig. 1A and supplementary tables S2D and S3A, Supplementary Material online). The sizes of the subgenome and the numbers of genes in Bhyb-ECI BhD and BhS were consistent with those of the respective progenitor species genomes (*B. stacei* Bsta-ECI and Bsta-ABR114, and *B. distachyon* Bdis-Bd21 and Bdis-Bd1-1, which were approximately 250 Mb and 275 Mb, respectively). The high-quality assembly of our Bhyb-ECI reference genome at the chromosome level, along with its comprehensive annotation (Fig. 1A), establishes a solid foundation for investigating the evolutionary mechanisms that underlie the recurrent speciation events of allotetraploid *B. hybridum* in diverse spatio-temporal scenarios and ecological contexts.

### Evolutionary Trajectories of Ancestral and Recent *B. hybridum* Allotetraploids Across Multiple Genomic Dimensions

To analyze recurrent allopolyploidizations of *B. hybridum* within an evolutionary framework, we enlarged our search to the BhD and BhS subgenomes of the other available *B. hybridum* allotetraploids (Bhyb-ABR113 and Bhyb-26), and to other *B. distachyon* (Bdis-Bd21 and Bdis-Bd1-1) and *B. stacei* (Bsta-ABR114 and Bsta-ECI) progenitor species genomes. Genome collinearity analysis detected high synteny between the genomic profiles of the Bhyb-ECI BhD and BhS subgenomes and their corresponding *Brachypodium* progenitor species' genomes and other allotetraploids' subgenomes (Fig. 1B). Next, we performed ortholog-clustering analysis using all genes of the studied *Brachypodium* genomes and the *O. sativa* genome (supplementary table S4, Supplementary Material online). This pangenome-level search rendered a total of 15,042 core ortholog gene clusters (OGCs) present in all genomes, 17,301 in all *Brachypodium* genomes, 19,745 in all S (*B. stacei*-type) (sub-)genomes and 19,420 in all D (*B. distachyon*-type) (sub-)genomes (supplementary fig. S3B, Supplementary Material online). In contrast, all ten *Brachypodium* (sub-)genomes had very few (sub-)genomes-specific dispensable OGCs (supplementary fig. S3B, Supplementary Material online) and genes compared with common OGCs (supplementary fig. S3C, Supplementary Material online). These results supported the conservative genome landscape at gene level among *B. hybridum*, *B. distachyon*, and *B. stacei*. Furthermore, a total of 12,481 single-copy ortho-homeologous genes (SCOGs) identified in *Brachypodium* and *Oryza* (supplementary fig. S3C, Supplementary Material online) were used to investigate the origins of eastern Mediterranean Bhyb-ECI and the other *B. hybridum* lineages through time-measured phylogenomic analysis.

**Fig. 2.**
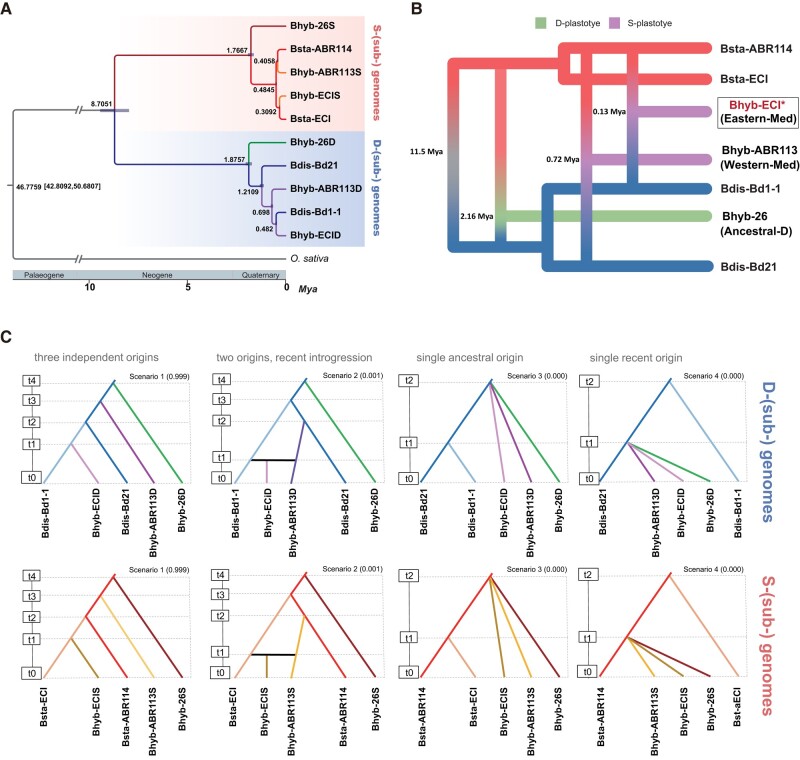
Phylogenomics, cross-bracing, and evolutionary model analyses. Phylogenomic analysis based on 12,481 nuclear SCOGs of *Brachypodium stacei* (Bsta-ECI and Bsta-ABR114), *B. distachyon* (Bdis-Bd1-1 and Bdis-Bd21), and *B. hybridum* (nuclear BhD and BhS subgenomes) of the eastern Mediterranean Bhyb-ECI and western Mediterranean Bhyb-ABR113 and Bhyb-26 allotetraploids, and *O. sativa* (see supplementary table S3, Supplementary Material online for more information). A) Dated Bayesian tree of all representative *Brachypodium* species genomes and subgenomes. Estimated nodal ages are indicated in black; *O. sativa* was used to root the tree. B) Inferred cross-bracing ages for the respective formations of the ancient (D-anc Bhyb-26) and recent Western Mediterranean (West-Med Bhyb-ABR113) and Eastern Mediterranean (East-Med Bhyb-ECI) *B. hybridum* clades (see coalescent-based SNP and AFLP package for phylogenetic analysis (SNAPP) tree computed from 30,000 filtered SCOG SNPs and used as baseline for the cross-bracing estimates, supplementary fig. S5, Supplementary Material online). All branches showed strong posterior probability support (1.0) in both topologies. C) Posterior probabilities of the four compared evolutionary scenarios on the alternative origins of *Brachypodium hybridum* allotetraploids estimated separately for the D and the S genomes/subgenomes through approximated Bayesian computation and RF methods. Scenario 1: three independent origins of ancestral (Bhyb-26), recent West-Mediterranean (Bhyb-ABR113), and very recent East-Mediterranean (Bhyb-ECI) allotetraploids; scenario 2: two independent origins of ancestral (Bhyb-26) and recent West-Mediterranean (Bhyb-ABR113) allotetraploids, followed by introgression of Bhyb-ABR113 with East-Mediterranean progenitors; scenario 3: single ancestral origins for the three allotetraploids; scenario 4: single recent origin for the three allotetraploids. Four thousand data sets per scenario were simulated for the training set and a forest of 2,000 random trees was generated for model choice. Posterior probabilities are indicated within parentheses for each scenario. Scenario 1 showed the highest posterior probability for both D and S genomes/subgenomes, supporting the three independent origins of *B. hybridum* allotetraploids, each from different progenitors.

A dated Bayesian chronogram (Fig. 2A) based on the 12,481 SCOGs and a secondary calibration for the *Brachypodium/Oryza* split inferred different divergence ages between the *B. distachyon* and *B. stacei* progenitor species lineages and their respective Bhd and BhS child lineages for each allotetraploid. The splits for the more ancestral D-anc Bhyb-26 and the more recently evolved West-Med Bhyb-ABR113 subgenomic lineages were estimated to have occurred 1.87–1.76 Ma and 1.21(0.69)−0.48 Ma, respectively, while the inferences for the East-Med Bhyb-ECI subgenomic lineages indicated relatively recent splits of 0.48–0.30 Ma (Fig. 2A). These age estimates were slightly older than those based on the distributions of non-zero synonymous substitutions per synonymous site (Ks) (Gaut et al. 1996) of ortho-homeologous genes in intergenomic syntenic blocks (Bhyb-ECID vs Bdis-Bd1-1, Ks_peak_ ∼ 0.0035, *t* = 0.27 Ma; Bhyb-ECIS vs Bsta-ECI, Ks_peak_ ∼ 0.0031, *t* = 0.24 Ma, *μ* = 6.5 × 10^−9^) (supplementary fig. S4, Supplementary Material online). We also performed pairwise-count of gene pairs that had no synonymous substitutions constructing two quadruple ortho-homeologous gene chains of D-(sub)genomes (Bhyb-ECID, Bhyb-ABR113D, Bdis-Bd1-1, Bdis-Bd21) and S-(sub)genomes (Bhyb-ECIS, Bhyb-ABR113S, Bsta-EC, Bsta-ABR114) based on ortho-homeologous genes in each genome set to test the closeness of genomic/subgenomic relationships. The D-genome-type contained 24,531 gene chains and S-genome-type 25,010; the pairwise-count tests indicated that Bhyb-ECID and Bhyb-ECIS have significantly more zero ks gene pairs with, respectively, Bdis-Bd1-1 and Bsta-ECI than with Bdis-Bd21 and Bsta-ABR114 (supplementary fig. S4, Supplementary Material online). These results supported the closeness of the Bhyb-ECI BhS and BhD subgenomes to the local East-Med Bsta-ECI and Bdis-Bd1-1 genomes, a finding consistent with the phylogenetic evidence (Fig. 2A).

A coalescence-based dating analysis performed with 30,000 filtered single nucleotide polymorphism (SNP) data from the SCOGs data set inferred similar divergence times for the splits of the ancestral Bhyb-26 subgenomic lineages (2.33 and 1.98 Ma) but more recent ages for those of Bhyb-ABR113 (0.89 and 0.55 Ma) and especially Bhyb-ECI (0.14 and 0.13 Ma) (supplementary fig. S5, Supplementary Material online). As the *B. hybridum* BhS and BhD splits corresponding to the same hybridization event showed nearly the same age for the ancestral event and close ages for the recent events, we used the cross-bracing approach that forces the split times of each parental genome to be contemporaneous. We obtained single-age estimates for the respective origins of D-anc Bhyb-26, which formed 2.16 Ma (95% Highest Posterior Density [HPD] 2.54–1.78), and of recent West-Med Bhyb-ABR113 (0.72 Ma; 95% HPD 0.88–0.56), that are relatively consistent though older than previous estimates (Gordon et al. 2020), and a very recent origin for the newly studied East-Med Bhyb-ECI (0.13 Ma; 95% HPD 0.24–0.02) (Fig. 2B). To further assess the maternal ancestor of the newly studied East-Med *B. hybridum* lineage we performed phylogenomic analysis based on whole plastome sequences of several *B. hybridum*, *B. distachyon* and *B. stacei* accessions. The plastome topology revealed that Bhyb-ECI as well as other individuals of this population and Israel showed a strongly supported *B. stacei*-type maternally inherited plastome, representing thus a new East-Med S-plastotype lineage that originated independently from the West-Med S-plastotype lineage (supplementary fig. S6A, Supplementary Material online). The plastome tree also suggested that the *B. hybridum* ECI plastomes were not inherited from local *B. stacei* ECI ancestors but from other eastern or western *B. stacei* sources, although the results were inconclusive due to low support of intraclade branches (supplementary fig. S6A, Supplementary Material online). Plastome lengths were longer and less variable in S-plastotypes than in D-plastotypes (136,289–136,333 bp for *B. stacei* and S-plastotype allotetraploids, and 134,991–135,423 bp for *B. distachyon* and D-plastotype allotetraploids) (supplementary fig. S6B, Supplementary Material online), and fell within the expected length ranges indicated previously (Sancho et al. 2018; Gordon et al. 2020).

To rule out possible introgressions of recent eastward-dispersed West-Med *B. hybridum* with eastern Mediterranean progenitor species that could falsify the putative third independent origin of the very recent East-Med *B. hybridum*, we tested alternative evolutionary scenarios for different origins of *B. hybridum* using filtered SCOG SNP data and coalescence approximated Bayesian computation and supervised machine learning methods. To test biologically meaningful scenarios, we split the full dataset into the D (sub)-genome and the S (sub)-genome subsets, considering that *B. hybridum* could backcross with one or the other progenitor species but not with both at the same time. We compared the same four alternative scenarios separately for the D and the S lineages with DIYABC-random forest (RF) (Fig. 2C and supplementary fig. S7 and supplementary table S5, Supplementary Material online). Scenario 1 (three independent origins of ancestral [Bhyb-26], recent West-Med [Bhyb-ABR113], and very recent East-Med [Bhyb-ECI] allotetraploids) was selected as the model with the highest number of votes and highest posterior probability over scenario 2 (two independent origins of ancestral and recent West-Med allotetraploids, followed by introgression of recent West-Med *B. hybridum* with eastern Mediterranean *B. distachyon* or *B. stacei* progenitors) and other less supported scenarios (scenario 3: single ancestral origins for the three allotetraploids; scenario 4: single recent origin for the three allotetraploids) (Fig. 2C and supplementary fig. S7 and supplementary table S5, Supplementary Material online).

**Fig. 3.**
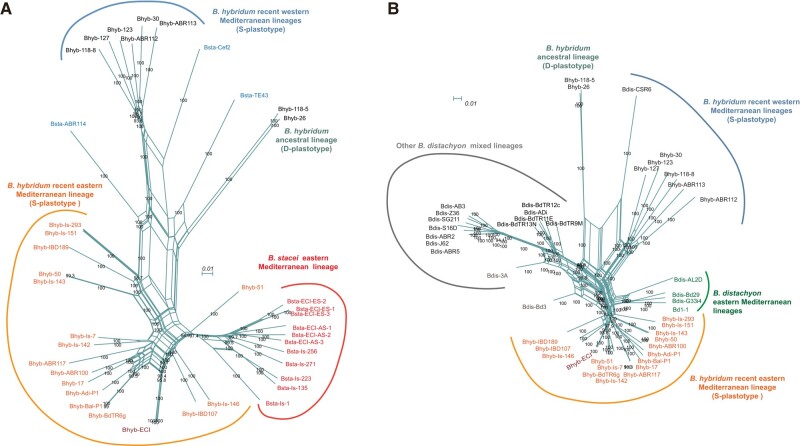
Population-level evolutionary analysis based on nuclear syntenic SNP data of *B. hybridum* (BhS subgenome) and its progenitor species *B. stacei* and of *B. hybridum* (BhD subgenome) and its progenitor species *distachyon.* Phylogenetic neighbor-networks of S genomes (*B. stacei* and *B. hybridum* BhS) (A) and of D genomes (*B. distachyon* and *B. hybridum* BhD) (B) computed with SplitsTree showing the respective divergence of *B. hybridum* and *B. stacei* and of *B. hybridum* and *B. distachyon* eastern Mediterranean populations from populations of these species from the western Mediterranean region. Only nodes with bootstrap >90 are shown.

To improve our understanding of the origins of the *B. hybridum* ECI population and other populations in Israel, we performed a population-level genomic study of their individuals and of available samples of other *B. hybridum* populations and of progenitor species *B. distachyon* and *B. stacei* populations from the eastern, central and western parts of the Mediterranean region (supplementary fig. S1, Supplementary Material online). Independent S-genome (*B. stacei* and *B. hybridum* BhS) and D-genome (*B. distachyon* and *B. hybridum* BhD) neighbor-networks constructed from syntenic SNP data showed the close relationships of the *B. hybridum* ECI and Israel lineages to *B. stacei* and *B. distachyon* lineages from Israel and the East-Med region, respectively, and their divergences from *B. hybridum* and progenitor species lineages from the central and western Mediterranean region (Fig. 3 and supplementary fig. S8, Supplementary Material online). The S-genome network retrieved close links between *B. hybridum* ECI and other populations from Israel to the *B. stacei* populations from the same settings (Fig. 3A and supplementary table S1, Supplementary Material online). The D-genome network recovered close ties of the *B. hybridum* Israel populations to *B. distachyon* East-Mediterranean populations from near Israel (Fig. 3B and supplementary table S1, Supplementary Material online). S-genomic relationships from multivariate PCA also discriminated the *B. hybridum* S-plastotype (plus *B. stacei*) eastern and western Mediterranean populations along PCA1 while the *B. hybridum* D-plastotype populations separated along PCA2 (supplementary fig. S8A, Supplementary Material online). Within the very closely related and recent S genomes/subgenomes population structure analysis detected three optimal genomic groups (best *K* = 3) differentiating the eastern *B. hybridum* populations from eastern *B. stacei* populations and western *B. stacei* + *B. hybridum* populations (supplementary fig. S8B, Supplementary Material online). Some individuals from eastern *B. hybridum* populations showed signatures of admixture with the other two genomic groups. The genetic diversity (*θπ*) value of East-Med *B. hybridum* (5.21e^−4^) population group was lower than that of the West-Med *B. hybridum* (6.01e^−4^) group (supplementary fig. S8C, Supplementary Material online), a result consistent with the more recent allotetraploidy of the former group (Fig. 2A). The pair-wised fixation index (*F*_ST_) between the three S-genome population groups showed significantly higher genomic divergence between the East-Med vs West-Med *B. hybridum* groups (0.528 and 0.547) than between East-Med *B. stacei* and East-Med *B. hybridum* (0.218) (supplementary fig. S8C, Supplementary Material online). These population scale results strongly support the in situ origin of the recently evolved East-Med *B. hybridum* lineage (S-plastotype) from local ancestors in this area (Fig. 3 and supplementary fig. S8, Supplementary Material online).

### Conservative Genomic Traits Between *B. hybridum* Subgenomes and Progenitor Genomes

To assess the genomic consequences after polyploidy, we performed comparative genomic analysis between Bhyb*-*ECI and its closest progenitor's genomes (Bsta-ECI and Bdis-Bd1-1). The GC contents of Bhyb-ECID and Bhyb-ECIS were 46.43% and 45.52%, respectively, which were similar than the GC content of the two progenitor species and other corresponding (sub-)genomes (46.42% and 45.43%, respectively) (supplementary fig. S9A, Supplementary Material online). Further analysis indicated that repetitive element content was greater in the two subgenomes of Bhyb-ECI than in the corresponding progenitor species' genomes, especially in regions near encoding genes (supplementary figs. S9B and C and supplementary table S6, Supplementary Material online), except for recent LTR Copia and Gypsy insertion burst detected in both subgenomes and in the corresponding progenitors' genomes (supplementary fig. S9D, Supplementary Material online). By comparing the whole Bhyb*-*ECI genome to the concatenated progenitor species genomes (Bsta-ECI and Bdis-Bd1-1), we found 486.58 Mb of collinear regions and 38.15 Mb of rearranged regions in the Bhyb*-*ECI subgenomes (supplementary fig. S9 and supplementary table S7, Supplementary Material online), consistent with the high syntenic conservation of the Bhyb*-*ABR113 genome detected previously (Gordon et al. 2020). These rearrangements included 12.82 Mb (*n* = 147) of inversions, 6.18 Mb (*n* = 1,539) of translocations, and 19.15 Mb (*n* = 8,444) of polymorphic duplications (supplementary fig. S10 and supplementary table S7, Supplementary Material online). At gene level, we found 27,171 orthologous gene pairs in Bhyb*-*ECID and Bdis-Bd1-1, 14,408 of which had no synonymous substitutions (Ks = 0, 53.02%), and 10,314 (37.95%) had identical sequences, whereas we detected 30,206, 17,780 (58.86%), and 13,372 (44.26%) gene pairs in similar Bhyb*-*ECIS and Bsta-ECI comparisons (supplementary table S8, Supplementary Material online). When we scrutinized the other allotetraploids, we found 27,873, 14,649 (52.55%), and 10,550 (37.85%) orthologous genes pairs in Bhyb-ABR113S and Bsta-ABR114, 30,371, 9,952 (32.7%), and 5,744 (18.91%) in Bhyb-ABR113D and Bdis-Bd21, 21,633, 1,279 (5.91%), and 393 (1.81%) in Bhyb-26S and Bsta-ABR114, and 22,741, 1,550 (6.81%), and 451 (1.98%) in Bhyb-26D and Bdis-Bd21 comparisons (supplementary table S8, Supplementary Material online).

### Low Occurrence of HEs and Similar Expression of Meiotic Genes in BhD and BhS Subgenomes of *B. hybridum* Allotetraploids

To encompass various spatio-temporal scenarios involving multiple origins of *B. hybridum*, we constructed two types of progenitor genomes: Ref-East (concatenated Bsta-ECI and Bdis-Bd1-1) and Ref-West (concatenated Bsta-ABR114 and Bdis-Bd21) (see supplementary fig. S11 and supplementary table S9, Supplementary Material online). When aligning *B. hybridum* samples from the eastern Mediterranean to the merged progenitor genomes (Ref-East and Ref-West), we observed a significantly lower number of outlier blocks with the Ref-East genome (supplementary fig. S11A and supplementary table S9D, Supplementary Material online). The two ancestral D-plastotype *B. hybridum* samples exhibited a similar and substantial number of putative deletion and duplication blocks when mapped to either the Ref-East or Ref-West merged genomes (supplementary fig. S11B and supplementary table S9D, Supplementary Material online). These findings highlight the crucial influence of selecting an appropriate reference progenitor genome for accurately identifying HEs (supplementary figs. S11A and C, Supplementary Material online). Consequently, we performed our analysis on an additional 21 East-Med *B. hybridum* samples using Ref-East genomes and 6 West-Med *B. hybridum* samples using Ref-West genomes to ensure reliable results. Only five reliable “HE with replacement” swaps were identified in the eastern Mediterranean *B. hybridum* samples when mapped to their closest Ref-East genomes (supplementary table S9A, Supplementary Material online); one of these swaps occurred in Bhyb-ECI and other 11 samples (supplementary fig. S11A and supplementary table S9A, Supplementary Material online). In contrast, four “HE with replacement” swaps were identified in western Mediterranean *B. hybridum* samples and two ancestral (D-anc) *B. hybridum* samples when using Ref-W as reference genome (supplementary tables S9B and C, Supplementary Material online), likely due to under-sampling of close progenitor genomes. Notably, no “reciprocal HE” events were found in any of the three *B. hybridum* lineages. Our analysis revealed that HE events were infrequent in Bhyb-ECI and the other studied allotetraploids (supplementary fig. S11 and supplementary table S9, Supplementary Material online), suggesting that the two duplicated subgenomes maintain stable bivalent chromosome pairing during meiosis.

Interestingly, we observed geographical variations in the depth patterns of outlier regions between the BhS subgenomes of western and eastern Mediterranean *B. hybridum* accessions and their respective *B. stacei* progenitor genomes. Specifically, the Bhyb-ABR113S subgenome exhibited a similar pattern of deletion regions with the Bsta-ABR114 genome when using Ref-East as the reference genome. However, this pattern differed (with the absence of deletions) when comparing the same chromosomal region in the Bsta-ECIS subgenome to its Bsta-ECI progenitor genome using the same reference genome (supplementary fig. S11D, Supplementary Material online). These results confirm the highly conserved genomic structure between the progenitor genomes and their respective descendant BhS subgenomes, further supporting the distinct evolutionary origins of the recent East-Med and West-Med S-plastotypes *B. hybridum* lineages.

The comparative analysis of the functional expression of meiotic *Ph1* and *Ph2* orthologs in the subgenomes of the studied *B. hybridum* allotetraploids revealed a balanced expression of *Ph2*-type genes from the BhD and BhS subgenomes in the leaf tissues of the D-anc Bhyb-26 allotetraploid (52.511 vs 44.134) and similarly low but equivalent values in the roots (2.7035 vs 2.6857) (supplementary table S10, Supplementary Material online). On the other hand, the expression of *Ph1*-type genes from both subgenomes exhibited only residual but similar values in these tissues. In the recent West-Med Bhyb-ABR113 allotetraploid the expression levels in BhD and BhS were low and equivalent for *Ph1*-type (1.8761 vs 1.4200) and *Ph2*-type (1.9465 vs 1.2292) genes in spikelet tissues and nearby absent in leaves. In the recent East-Med Bhyb-ECI allotetraploid, the overall expressions were generally low but balanced between the two subgenomes (Ph2-type genes in roots: 0.6940 vs 0.3946; Ph1-type genes in leaves: 0.5016 vs 0.1214, and roots: 0.2961 vs 0.5315) (supplementary table S10, Supplementary Material online).

### Lack of HEB and Evidence That Gene Expression Changes Contributed to Ecological Adaptations of *B. hybridum* Allotetraploids

**Fig. 4.**
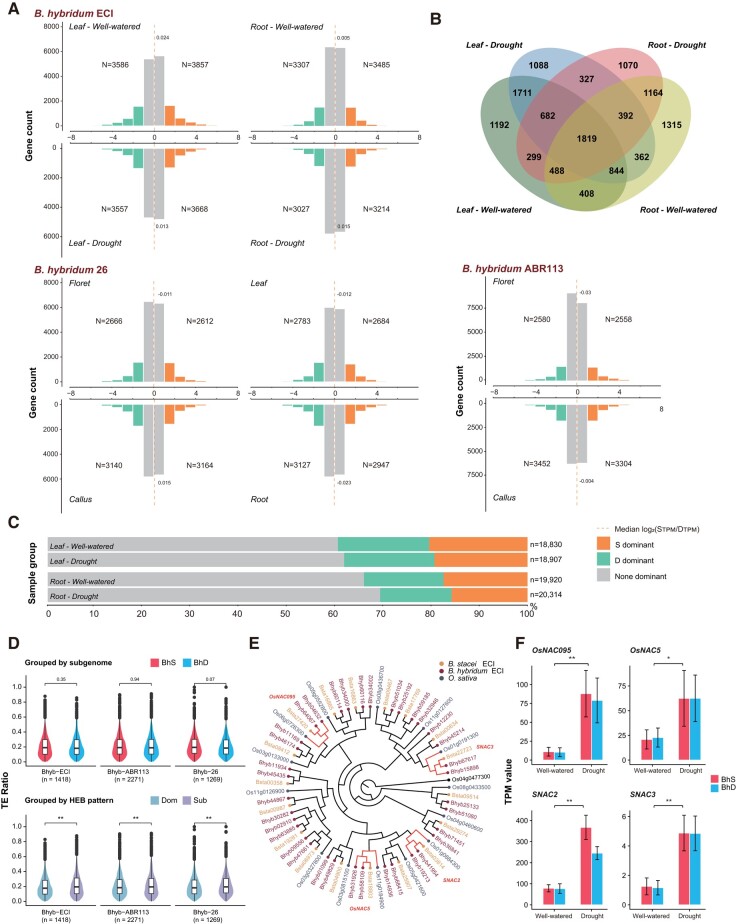
Unbiased homeologous gene expression in *Brachypodium hybridum*. A) Histograms showing the distribution of HEB of genes in the BhD and BhS subgenomes of *B. hybridum* Bhyb**-**ECI (leaf and root tissues of samples grown under well-watered vs drought conditions), *B. hybridum* Bhyb**-**ABR113 (callus and floret tissues of plants grown under control conditions), and *B. hybridum* Bhyb**-**26 (callus, floret, leaf, and root tissues of plants grown under control conditions). Log2 fold-changing (BhSTPM/BhDTPM) indicates the degree of HEB. Homeologous gene pairs were classified as biased toward BhS or BhD dominance. B) *B. hybridum* Bhyb**-**ECI: overlap of homeologous gene pairs that exhibit HEB between different tissue and treatment groups (leaf-well watered; leaf-drought; root-well watered; root-drought). C) Numbers and percentages of HEB genes from the four sample groups across the *B. hybridum* Bhyb-ECI gene expression atlas. D) Comparisons of TE density in genes in the homologous gene pair showed the same expression bias patterns in all case study groups in Bhyb-ECI, Bhyb-ABR113, and Bhyb-26, respectively. Grouped by subgenome (BhD and BhS) and grouped by expression bias type (Dom: dominant, Sub: submissive), **P-value < 0.01 (paired Wilcoxon test). E) Phylogenetic relationships and orthology prediction of abiotic stress-related NAC genes in Bhyb-ECI and Bsta-ECI using *O. sativa* as an outgroup. F) Significant differential gene expression of BhD and BhS homeologs of four representative NAC transcription factors in individuals grown under well-watered vs drought conditions. Values shown are TPM; *P-value < 0.05, **P-value < 0.01 (Wilcoxon test).

To investigate subgenome dominance patterns in Bhyb-ECI of *B. hybridum* and to compare it with its other allotetraploids (Bhyb-ABR113, Bhyb-26), we analyzed gene expression in leaf and root tissues of Bhyb-ECI samples under well-watered (control) and drought conditions (Fig. 4 and supplementary table S11, Supplementary Material online). We also examined gene expression in callus and floret tissues of Bhyb-ABR113 and in callus, floret, leaf and root tissues of Bhyb-26 under control conditions (Gordon et al. 2020; Scarlett et al. 2022). In contrast to many allotetraploid species that exhibit subgenome dominance, our pairwise comparisons showed that Log2(BhS_TPM_/BhD_TPM_) values were close to zero in all groups analyzed (Fig. 4A and supplementary table S11, Supplementary Material online). These results suggest that there was no apparent HEB between BhD and BhS homeologs in different tissues across the three ancestral and recent *B. hybridum* allopolyploid lineages (Fig. 4A), nor under any growth condition in Bhyb-ECI (Figs. 4B and C). At the gene level, we conducted a more comprehensive analysis of Bhyb-ECI, focusing on 1,819 out of 23,313 syntenic homeologous gene pairs between BhD and BhS that exhibited HEB in all groups studied (leaf-well watered, leaf-drought, root-well watered, and root-drought) (Fig. 4B). Among them, 1,418 gene pairs maintained consistent expression bias patterns across all four case study groups and did not show any subgenome dominance (supplementary table S12, Supplementary Material online).

This unbiased baseline provided an opportunity to investigate the cause of HEB, which is indicative of subgenome dominance and diploidization, as well as a potential constraint for stronger purifying selection (Cheng et al. 2018). Initially, we divided these consistently biased gene pairs into two subgenomic groups, BhD and BhS, and assessed the selection pressure by comparing the non-synonymous and synonymous substitution rates (Ka, Ks) of ortho-homeologous gene pairs with those of the corresponding progenitor species genomes. We found no evidence of significant differences (*P* = 0.53) between genes from the BhD and BhS subgenomes (supplementary fig. S12A, Supplementary Material online). Likewise, when we divided them based on expression levels, there were no significant differences (*P* = 0.44) in Ka/Ks between dominant genes (DGs) and their corresponding submissive genes (SGs) (supplementary fig. S12B, Supplementary Material online), suggesting that selection pressure is not a direct cause of HEB. Consistent with previous reports in *B. distachyon* (Gordon et al. 2017) and other plants (Hollister and Gaut 2009), gene expression levels in Bhyb-ECI showed a negative correlation with the density of nearby TEs in the whole genome background (*r*_Pearson_ = −0.30, *P* = 2.2e^−16^) (supplementary fig. S13, Supplementary Material online). Investigation of the TE density near these 1,418 stable gene pairs with biased expression in Bhyb-ECI did not detect a significant difference (*P* = 0.35) between the two subgenomes (Fig. 4D). However, there was a significant difference (*P* = 3.341^−7^) between DGs and their corresponding SGs (Fig. 4D).

Interestingly, when we examined the density of TEs near the orthologous genes of these gene pairs within the respective progenitor genomes (Bdis-Bd1-1 and Bsta-ECI), we discovered significant differences not only when grouping them by genomes but also when grouping them based on expression bias type (supplementary fig. S14, Supplementary Material online). A similar pattern of stable expression bias was also observed in Bhyb-ABR113 (*n*_genepair_ = 2,541) and Bhyb-26 (*n*_genepair_ = 1,478) (Fig. 4D and supplementary fig. S14, Supplementary Material online), indicating that it is a common pattern following recurrent allotetraploidy in *B. hybridum*. Furthermore, we observed that the difference values were predominantly centered around 0 in both recent *B. hybridum* lineages when comparing TE density near orthologous gene pairs between the progenitor genomes and subgenomes (supplementary fig. S13, Supplementary Material online). The conservation of both sequence and nearby TE density in the genes of stable biased gene pairs suggests that this HEB pattern may have been inherited from the progenitor genomes rather than emerging solely as a result of the allotetraploidy event. These findings are in line with the recent “nuclear chimera” model (Zhang et al. 2023), which posits that the two subgenomes of an allotetraploid maintain their gene expression patterns and regulatory networks until chromosomal rearrangement events disrupt the balance.

To validate this hypothesis, we investigated whether HEB maintained or altered the expression patterns of genes under drought conditions. Surprisingly, we observed a higher number of unbiased gene pairs in the leaf and root tissues of plants subjected to drought compared to those grown under well-watered conditions (Fig. 4C). This unexpected result contradicts previous findings in allotetraploid *Eragrostis* (VanBuren et al. 2020). Only 162 (1.69%) and 310 (3.38%) gene pairs exhibited converse dominance patterns in leaves and roots, respectively, between plants in well-watered and drought conditions (supplementary table S13, Supplementary Material online). In leaves, 4,894 genes (50.92%) with HEB maintained the same dominance pattern under both conditions, and in roots, this was observed for 3,553 genes (38.65%). Our findings suggest that approximately half of the homeologous gene pairs underwent changes in dominance patterns when plants were exposed to drought stress (supplementary table S13, Supplementary Material online), indicating independent gene regulation dynamics of the two subgenomes in response to drought.

We conducted an analysis of stress response of transcription factors belonging to the *NAC* gene family (You et al. 2015) in Bhyb-ECI. In *B. hybridum*, we identified two homeologous copies of most *NAC* genes, whereas *B. stacei* and *Oryza sativa* had only single copies (Fig. 4E). Interestingly, the majority of these genes were expressed in both leaf and root tissues of plants grown under both well-watered and drought conditions (supplementary fig. S15, Supplementary Material online), indicating constitutive expression regardless of the growth conditions. Additionally, we examined the expression of four well-known drought response genes (*ONAC095*, *OsNAC5*, *SNAC2*, and *SNAC3*) in Bhyb-ECI. We found that all of these genes were strongly induced in response to drought stress in both BhD and BhS homeologs (Fig. 4F).

## Discussion

### Genomic Stability and Subtle Evolutionary Novelties of the Recurrently Originated *B. hybridum* Allopolyploids: Most Traits Were Probably Inherited From Parents

Our study has made significant progress in understanding the genomic evolutionary history of the recurrently originated *B. hybridum* allopolyploid model system, building upon and reconciling previous findings (Gordon et al. 2020; Hasterok et al. 2022; Scarlett et al. 2022). Through comparative genomic and phylogenomic analyses using more comprehensive reference genomes of the three allotetraploid hybrids and their closely related progenitor species, we have confirmed their independent origins (Figs. 2 and 3). This suggests that post-WGD diploidization likely occurred immediately in these three allotetraploids, as there is no evidence of significant genomic rearrangements or subgenome dominance. The survival of a “successful” allopolyploid involves overcoming genomic dis-homeostasis, such as changes in chromosome numbers, unstable meiotic pairing, potential reconfiguration of gene expression regulatory networks (Comai 2005; Nieto Feliner et al. 2020; Deb et al. 2023) and initial competition with progenitor species (Te Beest et al. 2012). Our analyses have uncovered a third spontaneous origin of *B. hybridum* in the eastern Mediterranean region (East-Med lineage; Figs. 2 and 3 and supplementary figs. S1 and S5, Supplementary Material online), expanding the evolutionary timeline for the recurrent formation of this allotetraploid (Gordon et al. 2020; Scarlett et al. 2022). Moreover, backcrossing the allotetraploid with any of its diploid progenitors is highly challenging due to differences in ploidy levels, chromosome numbers, and postzygotic barriers (Catalan et al. 2016; Sancho et al. 2022). Our coalescence-RF analysis unequivocally rules out potential introgressions and other less reliable single origins, thus confirming the three separate origins (Fig. 2C). These results align with previous and current observations that have not detected intermediate chromosome numbers between those of *B. hybridum* and its diploid progenitors (Catalan et al. 2016), and with the different karyotype structure of the *B. stacei* and *B. distachyon* chromosomes (Gordon et al. 2020). Consequently, the potential occurrence of crossovers between “homeologous” S and D chromosomes within the *B. hybridum* nucleus is highly unlikely.

Our tests also provide support for the immediate amphidiploidy of the inter-specific annual hybrids, which suggests that they could reproduce within the same year and establish themselves as new allotetraploids (Sancho et al. 2022). The geographically distant locations of the two relatively recent West-Med (0.72 Ma) and very recent East-Med (0.13 Ma) *B. hybridum* S-plastotype allopolyploidization events (*B. stacei*-type maternal parents; Fig. 2 and supplementary figs. S5 and S6, Supplementary Material online), along with their proximity to current western and eastern Mediterranean populations of *B. stacei* and *B. distachyon* progenitors (Fig. 3 and supplementary fig. S8, Supplementary Material online) support parallel in situ speciation events. These hybrids likely originated from different local ancestors but resulted in the same allotetraploid species. The extensive distributions of these lineages across the circum-Mediterranean region (supplementary fig. S1, Supplementary Material online) suggest a rapid and successful adaptation to their original niches (Te Beest et al. 2012) and quick colonization of new niches after long-distance-dispersals (Scholthof et al. 2018). In contrast, the ancestral D-anc *B. hybridum* lineage is restricted to a small area in Southern Spain (2.16 Ma, Fig. 2 and supplementary fig. S5, Supplementary Material online), which overlaps with the distribution of the West-Med lineage (supplementary fig. S1, Supplementary Material online) and is likely its place of origin. However, the ancestral lineage's formation from a *B. distachyon*-type maternal parent (D-plastotype) and its old age, estimated to have preceded the divergence of the current progenitor species lineages (e.g. *B. distachyon*, ∼1 Ma, Sancho et al. 2018; Gordon et al. 2020; *B. stacei*, ∼0.22 Ma; unpublished data), make it an “orphan” allotetraploid with potentially extinct progenitor parents. Nonetheless, it exhibits similar phenotypic features to the recent S-plastotype allotetraploids. The different age estimates obtained for the D-anc and West-Med *B. hybridum* lineages in Gordon et al. (2020) (1.4 and 0.14 Ma, respectively) and in the current study (2.16 and 0.72 Ma; Fig. 2 and supplementary fig. S5, Supplementary Material online) may be affected by differences in sampling size and the use of more conserved SNP data set in this study. However, the age estimate of the very recent East-Med lineage (0.13 Ma; Fig. 2 and supplementary fig. S5, Supplementary Material online) is a consequence of selecting appropriate close progenitor D (Bdis-Bd1-1) and S (Bsta-EC) genomes (Fig. 3 and supplementary fig. S8, Supplementary Material online), which are unknown for the D-anc lineage and not available for the West-Med *B. hybridum* lineage (Figs. 2 and 3 and supplementary figs. S5 and S8, Supplementary Material online).

Despite being considered an important aspect of the evolution and establishment of many nascent plant allopolyploids (Mason and Wendel 2020; Deb et al. 2023), our results highlight the lack of significant HEs in the formation of the three *B. hybridum* lineages. We identified very few reliable cases of “HEs with replacement” (supplementary fig. S11 and supplementary table S9, Supplementary Material online; Scarlett et al. 2022), and no “reciprocal HE” events in all of these lineages. The absence of “reciprocal HEs” is consistent with the fact that these wild allotetraploids are not recent early-generation allotetraploids but rather late-generation allotetraploids (Figs. 2A and B and supplementary fig. S5, Supplementary Material online). In this context, the original “reciprocal HEs” would have undergone mosaicization and transformed into “HEs with replacement” over their evolutionary history (Deb et al. 2023). In contrast to the findings of Scarlett et al. (2022), which detected more genome rearrangements in the ancestral Bhyb-26 genome than in the more recent West-Med Bhyb-ABR113 genome, our genome-wide HE analyses indicate the overall lack of HE events in any of the three *B. hybridum* lineages (supplementary fig. S11 and supplementary table S9, Supplementary Material online). Our HE data do not support the hypothesis of gradual polyploid genome evolution in *B. hybridum* (Scarlett et al. 2022), but rather suggest immediate amplidiploidization of each allotetraploid in each temporal scenario, possibly due to the distinct structural karyotypes of two parental subgenomes (Gordon et al. 2020). Meiotic *Ph*1 and *Ph*2 ortholog expression analyses also support these findings (supplementary table S10, Supplementary Material online), indicating stable bivalent formation in all types of hybrids. Collectively, our assessments indicate that no major structural diploidization rearrangements occurred after the three recurrent allopolyploidization events (Fig. 1B and supplementary figs. S9 and S10 and supplementary tables S7, S9, and S10, Supplementary Material online), while some TE mobilization around genes in the BhD subgenome (supplementary fig. S9, Supplementary Material online) indicates that Bhyb-ECI, like the other *B. hybridum* lineages (Bhyb-ABR113; Bhyb-26; supplementary fig. S9, Supplementary Material online; Gordon et al. 2020; Scarlett et al. 2022), may have experienced transposon proliferations following allotetraploidization.

Interestingly, our analyses have revealed the potential for inaccurate detection of HEs referred to as “false positive outlier blocks” when aligning the *B. hybridum* genomes to inappropriate reference progenitor genomes (supplementary fig. S11A, Supplementary Material online). This issue becomes more pronounced in the “orphan” ancestral *B. hybridum* allotetraploid, which exhibits the highest number of questionable duplications and deletions when mapped to either the Ref-West or Ref-East merged progenitor genomes (supplementary fig. S11B, Supplementary Material online). Hence, it is plausible that the significant gene losses and TE proliferations previously detected in the ancestral D-anc Bhyb-26 compared to the recent West-Med Bhyb-ABR113 (Scarlett et al. 2022) could be attributed not only to gradual polyploid evolution but also to the lack of appropriate ancestral progenitor genomes for comparative genomics. Similarly, ambiguous evidence of HEs may have been misidentified in other plant allopolyploids (Edger et al. 2020) when close progenitor genomes are unknown (Liston et al. 2020) and comprehensive pangenomic sampling of progenitor genomes and hybrids is absent (Sancho et al. 2022). In contrast, the observed geographical variation in HE patterns between the western and eastern Mediterranean *B. hybridum* BhS subgenomes and their respective *B. stacei* progenitor genomes (supplementary fig. S11 and supplementary table S9, Supplementary Material online) confirms a highly conserved genomic structure in the progenitor genomes and their descendant *B. hybridum* subgenomes, suggesting that most of their genomic characteristics were probably inherited from the parents. Only subtle evolutionary novelties, such as TE turnovers, appear to have been acquired in each lineage (supplementary fig. S11 and supplementary table S9, Supplementary Material online).

### Subgenomic Background Rather Than HEB Drives the Adaptive Success of *B. hybridum*

In line with previous studies conducted on *B. hybridum* Bhyb-ABR113 (Gordon et al. 2020) and *B. hybridum* Bhyb-26 (Scarlett et al. 2022), the Bhyb-ECI genome does not exhibit any signs of subgenome dominance in both leaves and roots (Figs. 4A–C). However, it is worth noting that subgenome dominance does occur rapidly after the formation of allopolyploids, even after multiple generations (Yu et al. 2021). Contrary to the expectation that relaxed purifying selection may drive the diploidization process of the allopolyploid in a few generations or over time (Douglas et al. 2015; Deb et al. 2023), our observations indicate that selection pressure is not a direct cause of biased expression in a small group of genes (Fig. 4B). This holds true for both the comparison between BhS and BhD subgenomes and the comparison between dominant and submissive genes (supplementary fig. S12, Supplementary Material online). Furthermore, although our data indicate a difference in TE density surrounding these genes, which could be related to gene expression regulation and methylation (Lippman et al. 2004) and potentially lead to initial biased expression (Fig. 4D), further analysis within a progenitor-allotetraploid framework suggests that this difference between DGs and SGs may be inherited from the progenitors' genomes (supplementary fig. S14, Supplementary Material online) in Bhyb-ECI. This finding has also been confirmed in Bhyb-ABR113 and Bhyb-26 (Fig. 4D and supplementary fig. S13, Supplementary Material online) and may potentially be present in other plant genomes as well (Mason and Wendel 2020; Scarlett et al. 2022; Deb et al. 2023). By integrating our findings on the absence of biased expression in all three *B. hybridum* lineages and our analysis of stably biased expression gene pairs in an unbiased genomic background, we provide additional support for the “nuclear chimera” model (Zhang et al. 2023) from a different perspective.

The adaptive advantages of allopolyploids have been widely reported (Van de Peer et al. 2017). Previous studies have indicated that *B. hybridum* occupies a similar ecological niche to its two progenitor species within its native circum-Mediterranean range and has successfully established populations in other regions worldwide (Lopez-Alvarez et al. 2015; Catalan et al. 2016). However, while the distribution of *B. hybridum* overlaps with that of its progenitor *B. distachyon* species in higher latitudes and less arid regions of the western Mediterranean, it only coincides with the range of the warm-adapted *B. stacei* in lower latitudes and extremely arid conditions found in most of Israel (supplementary fig. S1, Supplementary Material online; Lopez-Alvarez et al. 2015; Penner et al. 2020). Interestingly, we discovered that drought stress can significantly alter the biased expression patterns in Bhyb-ECI (supplementary table S13, Supplementary Material online). Furthermore, the conserved subgenomic gene expression patterns of Bhyb-ECI suggest subtle gene loss, sub- or neo-functionalization, which would have maintained separate gene expression regulatory networks in the two subgenomes (Woodhouse et al. 2014). Our findings also suggest that there is an increased capacity for gene expression or regulation networks in response to drought stress (Fig. 4B), which could contribute to the environment adaptability of allotetraploid *B. hybridum.* Notably, the overexpression of drought-responsive NAC family genes in Bhyb-ECI under drought conditions, similar to those found in the arid habitat ECI-AS of Israel where *B. distachyon* is currently absent (Lopez-Alvarez et al. 2015; Mu et al. 2023), corresponds to BhD homeologs rather than to BhS homeologs (Fig. 4F and supplementary fig. S15, Supplementary Material online). These pieces of evidences suggest that a plausible drought tolerant *B. distachyon* ecotype, similar to those found in the East-Mediterranean region (Decena et al. 2021), may have been the potential parent of the current Bhyb-ECI and that its inherited genomic background, rather than biased expression, contributed to its adaptive success. The presence of multiple homeologous gene copies, generated through allotetraploidy and maintained throughout evolutionary history, may have enhanced the stress tolerance of *B. hybridum* in ECI, particularly in the dry AS environment and similar habitats throughout Israel and the Mediterranean region.

## Materials and Methods

### *Brachypodium hybridum* ECI Reference Genome

A new reference genome for *B. hybridum* from a sample collected at ECI (Bhyb-ECI; Lower Nahal Oren, Mount Carmel, Israel, 32°43′N; 34°58′E) was sequenced using a PacBio Sequel2 and Illumina HiSeq X Ten platform. In total 28.97** **Gb of circular consensus sequencing reads and 29.95 Gb of clean pair-end were generated for Bhyb-ECI. For Hi-C libraries, DNA was extracted from fresh leaves, chromatin was fixed with formaldehyde in the nucleus, and cross-linked DNA was digested with *DpnII*. Approximately 166.76 Gb of Hi-C raw reads were yielded using an Illumina HiSeq X (supplementary table S2A, Supplementary Material online).

Genome sizes were estimated using K-mer methods. K-mers counting program (KMC; v3) (Kokot et al. 2017) and FindGSE (v1.94) R package (Sun et al. 2018) were employed to generate K-mer distributions and calculate genome size (supplementary fig. S2A, Supplementary Material online). HiFi reads of the Bhyb-ECI genome were assembled using NextDenovo (v2.5.0) (Hu et al. 2023). Low-quality Hi-C reads were filtered using Fastp (v0.20.0) (Chen et al. 2018). Juicer (v1.9.9) (Durand et al. 2016) and 3D-DNA (v201008) (Dudchenko et al. 2017) were employed to anchor contigs to the 15 chromosomes of Bhyb-ECI (supplementary table S2C, Supplementary Material online). Genome assembly quality and completeness was assessed using Merqury (v1.3) (Rhie et al. 2020) and BUSCO (v3.0.2) (Simão et al. 2015) with the embryophya_odb10 dataset. LAI values were generated with LTR-retriever (v2.9.0) (Ou and Jiang 2018). In addition, SAMtools (v1.1) (Li et al. 2009) and Bamdst (https://github.com/shiquan/bamdst, v1.0.9) were applied for calculating mapping depth and coverage. Contig NG (Bradnam et al. 2013) was estimated using customized scripts.

The repetitive DNA sequences (tandem repeats and TEs) of the Bhyb-ECI genome were identified with TandemRepeatsFinder (TRF; v4.09b) (Benson 1999), RepeatModeler (v2.0) (Price et al. 2005), LTR-retriever (v2.9.0) (Ou and Jiang 2018), RepeatMasker (v4.0.7) (Chen 2004), and RepeatProteinMasker (included in the RepeatMasker package) based on the published Repbase (v20181026) (Jurka et al. 2005). All results were merged to non-redundancy repeat sequence annotation, and genome sequence in repeat region masked using Bedtools (v2.29.1) (Quinlan and Hall 2010). Insertion times of the most abundant Gypsy and Copia retrotransposon families of the *B. distachyon* (Bdis-Bd21 and Bdis-Bd1-1) and *B. stacei* (Bsta-ABR114 and Bsta-ECI) genomes, and *B. hybridum* (Bhyb-ECI, Bhyb-ABR113, and Bhyb-26) BhD and BhS subgenomes were calculated using LTR-retriever with automatic default options and the rice's mutation rate (1.3e^−8^).

The gene prediction strategy involved the integration of multiple methods (ab initio, homology-based, and transcriptomic). Augustus (v3.2.3) (Stanke et al. 2006) was utilized for ab initio gene prediction. For homology-based prediction, we collected genome data from representative grass species, including *B. distachyon* (Bdis-Bd21), *B. stacei* (Bsta-ABR114, Bsta-ECI) *B. hybridum* (Bhyb-ABR113, Bhyb-26) and *Oryza sativa* (GCF_001433935.1), and employed GeMoMa (v1.6.4) (Keilwagen et al. 2019) to search for orthologous gene structures by mapping protein sequences of these species to Bhyb-ECI. Both alignment-based and de novo-based methods were employed for transcriptomic-based gene predictions. For the alignment-based approach, HISAT2 (v2.2.1) (Kim et al. 2019) was applied to map RNA-Seq data to the sequenced genome. StringTie (v2.1.6) (Pertea et al. 2015) and Transdecoder (https://github.com/TransDecoder/TransDecoder) were employed to generate the gene predictions based on information from the alignment. In the de novo-based method, we used Trinity (v2.9.1) (Haas et al. 2013) and the Program to Assemble Spliced Alignments (PASA)-pipeline (v2.4.1) (Haas et al. 2003) to assemble transcripts and obtain gene coding regions of genomes. All the gene models predicted through these approaches were integrated with EvidenceModeler (v1.1.1) (Haas et al. 2008) and this step generated a final gene feature file for functional annotation and downstream analysis.

BUSCO (v3.0.2) (Simão et al. 2015) with the embryophya_odb10 dataset was used as a measure of gene model completeness. The functional information of gene model predictions was annotated using InterproScan (v5.36) (Jones et al. 2014). Blast (diamond blastp) program was utilized to search for homeologous candidates with three public databases, Swiss-Prot (Bairoch and Apweiler 2000), RefSeq non-redundant data base (NR) (Pruitt et al. 2007), and TrEMBL. Gene Ontology (GO) term annotations integrated results from InterProScan, the EggONG (v3.5) (Huerta-Cepas et al. 2019) and Blast2GO pipeline (v6.0) (Conesa et al. 2005). Transcription factors within the gene models were identified using iTAK (web version) (Zheng et al. 2016). The subgenomes of allotetraploid *B. hybridum* Bhyb-ECI were identified merging the two progenitor species genomes (*B. stacei* Bsta-ECI and *B. distachyon* Bdis-Bd1-1) and performing genome synteny analysis using whole-genome duplication integrated analysis (WGDi; v0.6.1) (Sun et al. 2022). The genome collinearity between all *Brachypodium* genomes (Bhyb-ABR113, Bhyb-26, Bhyb-ECI, Bsta-ABR114, Bsta-ECI, Bdis-Bd21, Bdis-Bd1-1) was performed with JCVI library (Tang et al. 2008).

### Phylogenomics, Cross-bracing Analysis and Testing of Alternative Origin Scenarios

For a more accurate inference of the evolutionary trajectory of the newly sequenced *B. hybridum* Bhyb-ECI genome, a phylogenetic framework was built containing: (i) the *B. stacei* reference genome Bsta-ABR114 and the new *B. stacei* Bsta-ECI genome generated from an individual collected in ECI (Mu et al. 2023); (ii) the *B. distachyon* reference genome Bdis-Bd21 and the potential closest *B. distachyon* genomes available within the pangenome data set of this second progenitor species (Bdis-Bd1-1); (iii) the two other reference genomes of *B. hybridum* (ancestral Bhyb-26 D-plastotype, recent Bhyb-ABR113 S-plastotype) (Gordon et al. 2020; Scarlett et al. 2022); and (iv) *O. sativa* used as out group. The phylogenetic scenario was constructed using their coding gene sequences. We obtained a high-quality, single-copy gene data set using OrthoMCL (v2.0.9) (Li et al. 2003), aligning the protein sequence of single-copy genes into a concatenated multiple sequence alignment (MSA) using MAFFT (v7.505) (Katoh and Toh 2008) and constructed a phylogenetic tree based on this MSA using Bayesian Evolutionary Analysis Sampling Trees (BEAST; v2.4.7) (General Time Reversible [GTR], gamma+ site heterogeneity, invariant sites, relaxed molecular clock, and Yule tree prior models, 10 million Mountain Chain Monte Carlo (MCMC) chain length, logging parameters every 500) (Bouckaert et al. 2014).

The homeologous gene chains (e.g. Bdis-Bd1-1-gene1, Bdis-Bd21-gene1, Bhyb-ECID-gene1, Bhyb-ABR113D-gene1, and Bhyb-26D-gene1) and their synonymous substitutions per synonymous site (Ks) were analyzed with WGDi (v0.6.1) (Sun et al. 2022) using the YN00 model. The divergence times between the two subgenomes of *B. hybridum* and those of its progenitor species for the three recurrent origins of the allotetraploid were estimated using the equation *T* = Ks/2*r*, where *r* stands for a divergence rate of 6.5 × 10^−9^ (Gaut et al. 1996).

We compared those clock-based estimations with coalescent-based estimations analyzed with SNAPP and a cross-bracing approach implemented by Gordon et al. (2020) to calculate the ages of the different origins of *B. hybridum*. We filtered the syntenic polymorphic positions of the concatenated genes for a reduced data set of all subgenomes of the three studied *B. hybridum* samples and selected clade members of *B. distachyon* and *B. stacei*, and run the SNAPP search using BEAST (v.2.4.7) (Bouckaert et al. 2014) imposing a normal prior distribution for a secondary age constrain at the *Brachypodium* crown node (11.6 ± 1.0 Ma), a 1/*x* distribution for clock rate and lambda (Yule model), and a uniform distribution for theta. The adequacy of parameters was checked using TRACER v.1.7 (Rambaut et al. 2018); most parameters showed effective sample size > 200, and a maximum clade credibility tree was computed after discarding 10% of the saved trees as burn-in. We followed the cross-bracing approach of McCann et al. (2018) adapted to SNAPP data by Gordon et al. (2020), using a cross-bracing normal distribution prior of 0 ± 0.02 (which enforces very low probability on trees that differ in node height and makes the age distributions of the cross-braced nodes become nearly congruent with respect to mean and shape) and running separate SNAPP searches for each allopolyploidization event (ancestral, recent West-Mediterranean, and recent East Mediterranean).

To test alternative evolutionary scenarios for different origins of *B. hybridum* we employed approximate Bayesian computation and supervised machine learning methods implemented in DIYABC-RF v.1.1.1-beta (Collin et al. 2021) for the separate *Brachypodium* D and S(sub)genomic filtered SCOG SNP data sets (7,780 and 40,199 SNP loci for the more and less divergent D and S genomic data sets, respectively). The DIYABC-RF approach enabled efficient discrimination among scenarios and estimation of the posterior probabilities. We compared four alternative scenarios for three (ancestral, recent, and very recent), two (ancestral and recent + introgression), single ancestral, and single recent origins of *B. hybridum* with the D and S datasets (Fig. 2C). For all scenarios, training sets were generated using 4,000 simulations per model, and prior distributions were uniform and set to default values. We identified the most likely scenario of each set using the RF module of DIYABC-RF, computing 2,000 random trees per model, as recommended in the manual, and the RF algorithm for model choice based on linear discriminant analysis.

### Population Genomics

The genomes of another 6 *B. hybridum* samples from the ECI population plus 6 additional samples from other localities from Israel were sequenced using the Illumina HiSeq X Ten platform and other representative circum-Mediterranean regions. *B. distachyon* (21 samples), *B. stacei* (13 samples) and *B. hybridum* (18 samples) accessions collected from NCBI database (supplementary supplementary fig. S1 and supplementary table S1, Supplementary Material online) were employed for population genomics analysis (Gordon et al. 2020; Scarlett et al. 2022; Mu et al. 2023).

The plastomes of *B. hybridum* Bhyb-ECI and Israel samples and several other accessions were assembled using Getorganelle (v1.7.1) (Jin et al. 2020). Whole plastome sequences (including large single copy region [LSC], SSC, IRa, and IRb regions) of *B. hybridum* and its progenitor species were aligned with multiple alignment using Fast Fourier Transform (MAFFT; v7.505) (Katoh and Toh 2008) and used to construct a maximum-likelihood tree with IQTREE (v1.6.12) (Nguyen et al. 2015) for inferring the maternal origins of the allotetrapoids.

The pair-end reads of each sample (from newly sequenced data and from NCBI data) were first filtered with Fastp (v0.20.0) to trim low-quality reads, and mapped to their corresponding genomes (Bsta-ECI for *B. stacei*, Bdis-Bd21 for *B. distachyon* and Bhyb-ECI for *B. hybridum*) using BWA-MEM2 (v2.2.1) (Vasimuddin et al. 2019) to generate unbiased sequence alignment files, and then the second alignment was filtered and sorted with SAMtools (v1.1) (Li et al. 2009). SNP from population samples of each species were called using “HaplotypeCaller” and “GenotypeGVCF” module and filtered using “VariantFiltration” (QualByDepth [QD] < 2.0 || FisherStrand (FS) > 200.0 || ReadPosRankSum < −20.0 and QD < 2.0 || FS > 60.0 || RMSMappingQuality (MQ) < 40.0 || MQRankSum < −12.5 || ReadPosRankSum < −8.0) module of Genome Analysis Toolkit (GATK; v3.8.1) (Depristo et al. 2011). Extra SNPs filtering steps were performed using custom Perl script with the following criteria: (i) labeling SNPs with non-information (./.) which had low (<1/3 of average chromosome) or high (>3-fold chromosome average) mapping depth; (ii) labeling SNPs with non-information (./.) showing quality scores ( Genotype Quality [GQ]) of genotypes < 10. Homeologous regions of the *B. stacei* S genomes and *B. hybridum* BhS subgenomes, and of *B. distachyon* D genomes and *B. hybridum* BhD subgenomes, were detected with Minimap2 (Li 2018) and then transformed to chain file using transanno (https://github.com/informationsea/transanno). The SNPs' coordinates of *B. stacei* samples were converted to those of the *B. hybridum* S subgenome, and those of *B. distachyon* samples to *B. hybridum* D subgenome using Crossmap (https://github.com/liguowang/CrossMap, v0.6.5). The VariantCallFormat (VCF) files were transformed to Fast-All (FASTA) format using customized Perl script, filtering sites with <80% information. Phylogenetic neighbor-networks were constructed separately for the S and D genomes/subgenomes using SplitsTree4 (Huson and Bryant 2006). The ADMIXTURE (100 bootstraps) (v1.3.0) (Alexander et al. 2009) and “smartpca” packages in EIGENSOFT (v7.2.1) (Patterson et al. 2006) were employed for population structure and Pirncipal Component Analysis (PCA) analysis. The Fixation statistic (*F*_ST_) and the nucleotide genetic diversities (*θπ*) were estimated using pixy (v1.2.7) (Korunes and Samuk 2021) with a 10 kb window size.

### HE Analysis

HE regions between the BhD and BhS subgenomes were identified following Chalhoub et al. (2014), and the HE regions were mainly detected based on mapping depth and sequence collinearity. We first detected whether different reference genomes affected the identification of HEs using two combined progenitor genome types, represented as Ref-East for the concatenated Bsta-ECI + Bdis-Bd1-1 genomes, and Ref-West for the concatenated Bsta-ABR114 + Bdis-Bd21 genomes. The syntenic collinearity blocks between progenitor genomes (Bsta-ECI vs Bdis-Bd1-1, and Bsta-ABR114 vs Bdis-Bd21) were generated by linking aligned regions with distances less than 20 kb identified with Minimap2 (v2.21) (Li 2018).

Using mapping depth approach, we performed analyses of the HEs pattern in the *B. hybridum* allopolyploids at the population scale intending to cover all spatio-temporal scenarios for the multiple origins of *B. hybridum*. The paired-end reads of each sample were mapped to these concatenated references genomes using BWA-MEM2 (v2.2.1), filtering the secondary alignments and sorting them using SAMTools (v1.1) to generate the Binary Alignment/Map (BAM) files. Then, the mapping depth was calculated with non-overlapping 10 kb window using Bamdst. The window had an outlier depth (within the 1.5–5 fold and 0–0.5 fold ranges of the corresponding chromosome average depth for each sample) which was used as threshold to detect the potential duplications and deletions windows. Adjacent windows were linked together if they had the same mark type. The regions spanning more than six windows (60 kb) and located in the two progenitor species genomes' syntenic collinearity blocks were considered as putative HE regions and transformed into coordinates in the *B. hybridum* genomes (Bhyb-ECI, Bhyb-ABR113, and Bhyb-26). The few detected HEs corresponded to “HE with replacement” events (reciprocal duplications/deletions in the respective homeologous chromosomes), while no “reciprocal HE” events (syntenic swapped regions between the respective homeologous chromosomes) (Mason and Wendel 2020; Deb et al. 2023) were detected in any of the three late-generation allotetraploids.

### Comparative Genome Analysis

The GC content, and the repetitive element content of total genome and near encoding genes were calculated through custom Perl scripts. The LTR insert time were estimated by LTR-retriever (v2.9.0) (Ou and Jiang 2018). The homeologous gene pair between subgenomes and corresponding progenitor genomes, and between the two subgenomes in three *B. hybridum* genomes were analyzed with WGDi (v0.6.1), and Ka and Ks values were obtained using WGDi (v0.6.1) with the YN00 model (v0.6.1) (Sun et al. 2022). The structural variations between subgenomes and corresponding progenitor genomes were identified by Nucmer (v4.0.0beta2) (Marçais et al. 2018) and SyRI (v1.6) (Goel et al. 2019). The *Ph1* (Martín et al. 2021) and *Ph2* (Serra et al. 2021) orthologs in *B. hybridum* genomes were identified on Basic Local Alignment Search Tool (BLAST) (blastp) searching homologous proteins against hexaploid bread wheat (*Triticum aestivum* L., AABBDD 2*n* = 6*x* = 42) (*Ph*1 wheat homologs were located in chromosomes 3A, 3B, and 3D).

### Drought Stress Experiment, Transcriptome Sequencing and Gene Expression Analysis

A drought stress experiment was performed with three biological replicates of a *B. hybridum* ECI-AS sample, aiming to elucidate if its BhS subgenome was dominant over its BhD subgenome under strong soil water deficit conditions, similar to those of its native arid ECI AS site, and searching for drought-tolerance genes. Seeds from the individual samples were germinated and grown for 14 h light/10 h dark cycles and a constant temperature of 23 ± 2 °C and then plants were assigned to two treatment groups, drought in which water was withheld for one week, and control in which plants were watered every two days for the duration of the experiment. Leaf and root samples from control and drought-treated plants were collected for transcriptomic analysis. Total RNA was extracted from each sample using the QIAGEN RNeasy plant mini kit, and sequenced using a DNBseq-T7 system following RNA library construction with oligo dT methods. The transcriptomic data of the ancestral D-anc Bhyb-26 (SRR20045864, SRR20045873, SRR20045874, and SRR20045875) and recent West-Med Bhyb-ABR113 (SRR4094443, SRR4094444, SRR4094445, SRR4094446, SRR11836559, and SRR11836560) were collected from National Center for Biotechnology Information (NCBI). Transcriptome analysis was conducted using the “HISAT2-Stringtie-DESeq” pipeline (Conesa et al. 2016). RNA-Seq reads were mapped to the corresponding genome with HISAT2 (v2.2.1) (Kim et al. 2019) and gene expression abundances (transcripts per kilobase per million mapped reads, TPM) were calculated using StringTie (v2.1.6) (Pertea et al. 2015).

### HEB Analysis in *B. hybridum* Genomes

HEB was performed through syntenic gene pairs analysis (Yang et al. 2016) in the East-Med Bhyb-ECI genome (two treatment conditions and two tissues), the ancestral D-anc Bhyb-26 (one treatment condition and four tissues) (Scarlett et al. 2022) and recent West-Med Bhyb-ABR113 (one treatment condition and two tissues) (Gordon et al. 2020) genomes. Gene pairs between the BhS and BhD subgenomes of the three types of allotetraploids were identified with WGDi. After removing gene pairs with TPM values < 1 in all samples, differentially expressed gene pairs (with >2-fold TPM differences) were regarded as expression-biased gene pairs, exhibiting either BhS or BhD dominance. The gene in each pair with the higher expression level was classified as a DG, the other gene as a SG, and the two genes included in non-dominance gene pairs were regarded as neutral genes. All of analysis were performed with custom Perl scripts.

### Statistical Analysis

GO enrichment analysis was performed with GOATools (v1.1.6) (Klopfenstein et al. 2018) with Benjamini-Hochberg procedure (BH) correction methods (Benjamini and Hochberg 1995). R (v4.0; https://www.r-project.org/) utilities were employed for statistic test in this study, and the R package ggplot2 (v3.3.5) (https://ggplot2.tidyverse.org/) was used to visualize the results.

## Supplementary Material

msad259_Supplementary_Data

## Data Availability

Data for the empirical data analysis and scripts and data used to run the genomic and evolutionary analyses are deposited at public database: the sequencing data were deposited at NCBI database (https://www.ncbi.nlm.nih.gov/), and the project number is PRJNA791713. The genome assembly and main script used in analysis were uploaded at Github (https://github.com/Axolotl233/Brachypodium_hybridum-ECI-genome).
