# Supplementary material for: Subgenomic Stability of Progenitor Genomes During Repeated Allotetraploid Origins of the Same Grass Brachypodium hybridum
Source: Mol Biol Evol. 2023 Nov 24;40(12):msad259. doi: 10.1093/molbev/msad259 (PMC10708906; doi:10.1093/molbev/msad259)
Supplement: msad259_Supplementary_Data [file msad259_supplementary_data.pdf]

Supplementary Tables: *Brachypodium hybridum*

Table S1. Sampling of *Brachypodium hybridum*, *B. stacei* and *B. distachyon* accessions used in this study. (*) reference genomes, (♦) progenitor species genomes.

Accession	*Species*	Latitude	Longitude	Locality	Resource	Plastome type
Bhyb-ABR113 (*)	*B. hybridum*	38.738	-9.463	Portugal	Gordon et al 2020	S-plastotype
Bhyb-26 (*)	*B. hybridum*	38.391	-3.370	Spain	Gordon et al 2020	D-plastotype
Bhyb-ECI-AS (*) (7 samples)	*B. hybridum*	32.710	34.970	Israel	This study	S-plastotype
Bsta-ECI (*) (6 samples)	*B. stacei*	32.710	34.970	Israel	Mu et al 2023	S-plastotype
Bhyb-ABR100	*B. hybridum*	30.398	50.632	Iran	Scarlett et al 2022	S-plastotype
Bhyb-ABR112	*B. hybridum*	41.393	9.159	France	Scarlett et al 2022	S-plastotype
Bhyb-ABR117	*B. hybridum*	31.560	65.330	Afghanistan	Scarlett et al 2022	S-plastotype
Bhyb-Adi-P1	*B. hybridum*	37.771	38.352	Turkey	Scarlett et al 2022	S-plastotype
Bhyb-Bal-P1	*B. hybridum*	37.880	38.885	Turkey	Scarlett et al 2022	S-plastotype
Bhyb-BdTR6g	*B. hybridum*	39.754	33.537	Turkey	Gordon et al 2020	S-plastotype
Bhyb-IBD-107	*B. hybridum*	35.400	31.550	Israel	Scarlett et al 2022	S-plastotype
Bhyb-IBD-189	*B. hybridum*	34.960	32.570	Israel	Scarlett et al 2022	S-plastotype
Bhyb-7	*B. hybridum*	31.370	34.820	Israel	This study	S-plastotype
Bhyb-17	*B. hybridum*	30.611	51.604	Iran	Scarlett et al 2022	S-plastotype
Bhyb-30	*B. hybridum*	37.229	-7.199	Spain	Gordon et al 2020	S-plastotype
Bhyb-50	*B. hybridum*	32.047	34.940	Israel	Scarlett et al 2022	S-plastotype
Bhyb-51	*B. hybridum*	31.494	34.690	Israel	Scarlett et al 2022	S-plastotype
Bhyb-118-5	*B. hybridum*	36.751	-2.121	Spain	Gordon et al 2020	D-plastotype
Bhyb-118-8	*B. hybridum*	36.900	-2.080	Spain	Gordon et al 2020	S-plastotype
Bhyb-123	*B. hybridum*	38.225	-2.738	Spain	Gordon et al 2020	S-plastotype
Bhyb-127	*B. hybridum*	37.724	-2.977	Spain	Gordon et al 2020	S-plastotype

Bhyb-142	*B. hybridum*	30.330	34.910	Israel	This study	S-plastotype
Bhyb-143	*B. hybridum*	32.040	34.940	Israel	This study	S-plastotype
Bhyb-146	*B. hybridum*	32.930	35.450	Israel	This study	S-plastotype
Bhyb-151	*B. hybridum*	33.070	35.190	Israel	This study	S-plastotype
Bhyb-293	*B. hybridum*	33.250	35.670	Israel	This study	S-plastotype
Bsta-ABR114 (*♦)	*B. stacei*	38.678	1.403	Spain	Gordon et al 2020	-
Bsta-ECI (*♦) (6 samples)	*B. stacei*	32.710	34.970	Israel	Mu et al 2023	-
Bsta-Cef2	*B. stacei*	38.030	14.020	Italy	NCBI (ERR483544)	-
Bsta-TE4.3	*B. stacei*	28.183	-17.183	Spain	Gordon et al 2020	-
Bsta-1	*B. stacei*	35.400	31.550	Israel	Mu et al 2023	-
Bsta-135	*B. stacei*	34.850	31.550	Israel	Mu et al 2023	-
Bsta-223	*B. stacei*	34.950	31.880	Israel	Mu et al 2023	-
Bsta-256	*B. stacei*	34.960	32.570	Israel	Mu et al 2023	-
Bsta-271	*B. stacei*	35.220	32.910	Israel	Mu et al 2023	-
Bdis-Bd21 (*♦)	*B. distachyon*	33.761	44.403	Iraq	Gordon et al 2020	-
Bdis-Bd1-1 (♦)	*B. distachyon*	39.191	27.608	Turkey	Gordon et al 2020	-
Bdis-AB3	*B. distachyon*	39.17	-1.96	Spain	Gordon et al 2020	-
Bdis-ABR2	*B. distachyon*	43.60	3.26	France	Gordon et al 2020	-
Bdis-ABR5	*B. distachyon*	42.57	-0.56	Spain	Gordon et al 2020	-
Bdis-Adi	*B. distachyon*	37.77	38.35	Turkey	Gordon et al 2020	-
Bdis-AL2D	*B. distachyon*	41.10	20.01	Albania	Gordon et al 2020	-
Bdis-Bd3	*B. distachyon*	33.76	44.40	Iraq	Gordon et al 2020	-
Bdis-Bd29	*B. distachyon*	44.51	33.55	Ukraine	Gordon et al 2020	-
Bdis-BdTR9M	*B. distachyon*	40.08	31.33	Turkey	Gordon et al 2020	-
Bdis-BdTR11E	*B. distachyon*	37.80	41.77	Turkey	Gordon et al 2020	-

Bdis-BdTR12c	*B. distachyon*	39.74	34.65	Turkey	Gordon et al 2020	-
Bdis-BdTR13N	*B. distachyon*	36.76	44.53	Turkey	Gordon et al 2020	-
Bdis-CSR6	*B. distachyon*	37.85	14.70	Italy	Gordon et al 2020	-
Bdis-G33i4	*B. distachyon*	41.75	45.21	Georgia	Gordon et al 2020	-
Bdis-Gaz	*B. distachyon*	37.13	37.39	Turkey	Gordon et al 2020	-
Bdis-J62	*B. distachyon*	38.03	-2.86	Spain	Gordon et al 2020	-
Bdis-S16D	*B. distachyon*	42.19	0.10	Spain	Gordon et al 2020	-
Bdis-SG211	*B. distachyon*	41.43	-3.66	Spain	Gordon et al 2020	-
Bdis-Z3_6	*B. distachyon*	40.97	-1.65	Spain	Gordon et al 2020	-
Bdis-3A	*B. distachyon*	41.17	44.88	Armenia	Gordon et al 2020	-

Table S2. Characteristics of the *Brachypodium hybridum* (Bhyb-ECI) reference genome from Evolution Canyon I, Israel (ECI).

A) Sequencing statistics

**Method**	**Library type**	**Reads number**	**Data size (Gb)**	**Mean read length (bp)**	**Read N50 (bp)**
Illumina (clean data)	Paired	199,970,546	29.95	150	-
Pacbio Hifi	Single	1,532,383	28.97	18, 909	18,827
Hi-C (clean data)	Paired	1,111,734,146	166.76	150	-

B) Genome assembly parameters of *B. hybridum* Bhyb-ECI local reference genome.

**Genome feature**	***B. hybridum*** **Bhyb-ECI**
Total length of contigs (Mb)	545.21
Total length of assemblies (Mb)	527.87
Gap number	38
Number of contigs	159
Contig N50 (Mb)	18.70
Number of scaffolds	15
Scaffold N50 (Mb)	30.61
LAI	12.43
GC content (%)	46.08
Percentage of anchoring (%)	96.81

C) Summary of Hi-C mapping parameters

	B. hybridum **Bhyb-ECI**	
	**Read number**	**Percentage (%)**
Total read pairs	552,501,929	100
Mapped read pairs	486,591,312	88.07
Uniquely mapped read pairs	350,089,833	63.36
Valid interaction read pairs	213,043,469	38.55

D) Chromosome-level information

Chromosome	Length (nts)	No. scaffolds
BhD01	75,243,273	5
BhD02	61,176,761	1
BhD03	60,232,616	4
BhD04	49,870,743	5
BhD05	29,305,787	2
BhS01	31,591,000	3
BhS02	30,008,228	2
BhS03	27,853,730	4
BhS04	26,551,000	4
BhS05	25,112,270	3
BhS06	23,254,000	3
BhS07	22,674,000	3
BhS08	22,066,000	1
BhS09	21,855,730	9
BhS10	21,064,497	4
Total	527,878,635	53

E) Summary of genome assembly validation and statistics

	Values
BUSCOs (%)	98.9
Complete and single-copy BUSCOs (%)	12.5
Complete and duplicated BUSCOs (%)	86.4
Fragmented BUSCOs (%)	0.1
Missing BUSCOs (%)	1.0
Mapping statistics	
Fraction of Mapped Illumina Data (%)	99.87
Fraction of Properly read pairs Mapped (%)	99.22
Regions of Coverage > 0x (%)	98.94
Regions of Coverage ≥ 10x (%)	98.56
K-mer	
Base call accuracy (QV)	38.79
Completeness	99.38

Table S3. Gene predictions and statistics in the assembled *B. hybridum* Bhyb-ECI genome from Evolution Canyon I, functional annotations and TF predictions.

A) Gene predictions comparisons of the *B. hybridum* Bhyb-ECI genome to those of the reference genomes of *B. stacei* Bsta-ABR114, *B. distachyon* Bdis-Bd21, Bdis-Bd1-1, and *B. hybridum* ABR113 v. 1.1, Bhyb26 (JGI; Phytozome, https://phytozome-next.jgi.doe.gov/), and of *Oryza sativa* subsp. japonica, (NCBI_Assembly: GCF_001433935.1).

**Species**	**Gene Predicted**	**Average Gene Length (bp)**	**Average CDS Length (bp)**	**Average Exons per Gene**	**Average Exon Length (bp)**	**Average Intron Length (bp)**	**Complete BUSCO (%)**
**Bhyb-ECI**	72,685	2417.03	1110.19	4.43	250.33	383.75	99.8
**Bhyb-ECIS**	33,766	2507.74	1137.23	4.55	249.59	385.37	-
**Bhyb-ECID**	38,330	2344.52	1087.28	4.34	250.83	376.04	-
Bsta-ABR114	29,898	3334.01	1197.60	4.76	251.26	393.98	99.7
Bdis-Bd21	34,310	3373.23	1117.39	4.39	254.36	403.07	99.5
Bdis-Bd1-1	32,667	3324.53	1158.34	4.43	261.31	401.06	99.4
Bhyb-26	53,836	3549.64	1211.26	4.77	253.66	397.84	99.5
Bhyb-ABR113	70,160	2973.68	1102.12	4.4	249.98	390.88	99.6
O. sativa	37,869	2986.76	982.71	3.74	262.43	426.67	99.7

B) Functional annotation of the predicted genes for the *B. hybridum* Bhyb-ECI genome

	**Database**	***B. hybridum* Bhyb-EC**	
		**Number**	**Percentages (%)**
Total		72,685	100
	NR	65,310	89.9
	InterPro	63,507	87.3
Annotated	Swissport	42,295	58.1
	TrEMBL	67,624	93.0
	EggNOG	56,878	78.3
Unannotated		2,164	3.0

C) Transcription factor (TF) prediction in the *B. hybridum* Bhyb-ECI genome

TF family	Number
Alfin-like	18
AP2/ERF-AP2	50
AP2/ERF-ERF	258
AP2/ERF-RAV	8
B3	106
B3-ARF	53

BBR-BPC	6
BES1	14
bHLH	259
bZIP	170
C2C2-CO-like	19
C2C2-Dof	59
C2C2-GATA	62
C2C2-LSD	11
C2C2-YABBY	16
C2H2	234
C3H	115
CAMTA	14
CPP	18
CSD	8
DBB	8
DBP	10
DDT	14
E2F-DP	20
EIL	12
FAR1	202
GARP-ARR-B	15
GARP-G2-like	97
GeBP	30
GRAS	120
GRF	24
HB-BELL	28
HB-HD-ZIP	76
HB-KNOX	21
HB-other	25
HB-PHD	6
HB-WOX	26
HRT	2
HSF	50
LFY	2
LIM	11
LOB	53
MADS-MIKC	68
MADS-M-type	89
MYB	245
MYB-related	122
NAC	258

NF-X1	4
NF-YA	14
NF-YB	29
NF-YC	28
OFP	62
PLATZ	31
RWP-RK	30
S1Fa-like	2
SBP	34
SRS	12
STAT	2
TCP	45
Tify	31
Trihelix	56
TUB	24
ULT	2
VOZ	4
Whirly	4
WRKY	174
zf-HD	44
All	3,764
Percentage of total gene predictions	5.1%

D) Prediction of repetitive elements in the assembled *B. hybridum* Bhyb-ECI genome

	Bhyb-ECI		Bhyb-ABR113		Bhyb-26	
	**Repeat Size (bp)**	**Percent of genome (%)**	**Repeat Size (bp)**	**Percent of genome (%)**	**Repeat Size (bp)**	**Percent of genome (%)**
**TRF**[1]	22,560,841	4.27	9,331,564	1.83	16,321,528	3.09
**RepeatMasker**	124,567,075	23.6	113,381,277	22.27	129,262,638	24.46
**RepeatProteinMask**	62,087,001	11.38	55,743,136	10.95	65,430,960	12.38
De novo	209,301,255	39.48	180,558,818	35.46	203,211,042	38.45
**Total**	219,622,865	41.6	194,648,738	38.13	219,402,990	41.51

Table S4. Summary of nuclear gene family information of genes used in the phylogenomic analysis of *B. hybridum* (Bhyb-ECI, Bhyb-ABR113, Bhyb-26), progenitor species *B. stacei* (Bsta-ECI, Bta-ABR114) and *B. distachyon* (Bdis-Bd21, Bdis-Bd1-1), and the outgroup *Oryza sativa*. Homeologous genes of allotetraploid *B. hybridum* are separated into their respective *B. distachyon*-type (BhD) and *B. stacei*-type (BhS) subgenomes.

Species	No. genes used	No. genes clustered	percentage
B. distachyon (Bd21)	34,310	31,706	92.41%
B. distachyon (Bd1-1)	32,667	28,701	87.85%
B. stacei (ECI)	32,951	30,985	94.03%
B. stacei (ABR114)	29,898	28,571	95.56%
B. hybridum ECID	38,330	35,000	91.31%
B. hybridum ECIS	33,766	32,015	94.81%
B. hybridum Bhyb26D	27,252	24,130	88.54%
B. hybridum Bhyb26S	26,522	23,312	87.89%
B. hybridum ABR113D	37,711	34,904	92.55%
B. hybridum ABR113S	32,449	30,604	94.31%
Oryza sativa	28,577	23,225	81.27%

Table S5. Inputs and results of tested evolutionary scenarios for alternative origins of *B. hybridum* using filtered SCOG SNP data and coalescence and supervised machine learning methods implemented in DIYABC Random Forest v1.1.1. Scenarios for the separate *Brachypodium* D and S (sub)-genomes datasets are described in the main text and shown in fig 2C.

(**A**) Values of demographic and historical parameters used for each of the four scenarios of each D and S (sub)-genomic datasets. t1-t4, time; ra, admixture rate; N, effective population size. All parameters were set to default priors; each prior value was drawn from a uniform distribution.

Scenario	t1	t2	t3	t4	ra	N1-N5
1	10 – 100000	10 – 100000	10 – 100000	10 – 100000	-	10-100000
2	10 – 100000	10 – 100000	10 – 100000	10 – 100000	0.05-0.95	10-100000
3	10 – 100000	10 – 100000	-	-	-	10-100000
4	10 – 100000	10 – 100000	-	-	-	10-100000

(**B**) Scenario choice inferred by DIYABC-RF. Mean number of votes and posterior probability of each scenario using out-of-bag estimators for each scenario with DIYABC-RF. Values of the best scenario (scenario 1) are highlighted in bold.

(sub) genome	Scenario	Votes	Posterior probability
	**1**	**0.540**	**0.999**
**D**	2	0.348	0.001
	3	0.080	0.000
	4	0.032	0.000
	**1**	**0.683**	**0.999**
**S**	2	0.294	0.001
	3	0.009	0.000
	4	0.014	0.000

Table S6. Summary of repetitive elements found in the assembled *B. hybridum* Bhyb-ECIS and Bhyb-ECID subgenomes and those of other allotetraploids (Bhyb-26, Bhyb-ABR113) with respect to those of progenitor species genomes *B. stacei* (Bsta-ECI, Bsta-ABR114) and *B. distachyon* (Bdis-Bd1-1, Bdis-Bd21). The first column of each genome shows the length(bp) of genome, and the second shows the percentage (%) of genome covered by repeat type.

S-(sub)genome

	**Bsta- ECI**		**Bhyb-ECIS**		**Bsta-ABR114**		**Bhyb-ABR113S**		**Bhyb-26S**	
**SINE**	611,446	0.238	670,923	0.266	436,828	0.19	757,061	0.316	712,663	0.286
**LINE**	7,583,328	2.954	6,993,788	2.775	6,785,199	2.9	7,613,984	3.174	8,909,941	3.578
L1	7,492,395	2.919	6,919,913	2.746	6,756,750	3.21	7,501,231	3.127	7,918,282	3.18
Other	210,321	0.08	123,201	0.04	29,953	0.01	212,660	0.089	1,130,602	0.454
**LTR**	46,478,386	18.106	47,721,925	18.934	35,045,130	14.97	37,851,924	15.778	44,308,052	17.794
Copia	16,894,953	6.581	17,774,800	7.052	10,043,204	4.29	11,661,864	4.861	14,175,527	5.693
Gypsy	29,297,286	11.413	30,067,048	11.929	23,934,981	10.22	25,539,628	10.646	29,039,923	11.662
Other	4,543,886	1.77	5,862,393	2.326	1,496,957	0.64	1,124,123	0.469	1,583,653	0.636
**DNA**	18,532,599	7.219	18,227,285	7.232	16,158,902	6.9	18,875,549	7.868	21,005,337	8.436
CMC-EnSpm	3,537,181	1.378	3,535,599	1.403	2,786,276	1.19	3,453,264	1.439	3,670,355	1.474
hAT-Ac	1,020,941	0.398	1,001,165	0.397	897,819	0.38	1,118,785	0.466	1,349,786	0.542
hAT-Tip100	443,859	0.173	603,652	0.24	571,236	0.24	678,763	0.283	850,476	0.342
MuDR	3,979,100	1.55	3,727,806	1.479	3,313,096	1.42	3,874,622	1.615	4,488,849	1.803
PIF-Harbinger	2,780,739	1.083	2,673,700	1.061	2,724,305	1.01	2,723,287	1.135	2,943,595	1.182
Other	7,339,227	2.859	6,984,026	2.771	6,325,645	2.7	7,745,344	3.229	8,595,658	3.452
**Transposable elements(TEs) total**	73,205,759	28.52	73,613,921	29.21	58,426,059	24.95	65,098,518	27.136	74,935,993	30.094
**Satellites**	126,999	0.049	163,071	0.065	14,724	0.01	194,999	0.081	153,019	0.061

**Simple_repeats**		19,846,896	7.731	16,959,857	6.729	5,427,411	2.32	6,182,325	2.577	10,356,633	4.159
**Unclassified**		29,067,671	10.38	27,799,342	11.03	16,443,949	7.02	19,763,755	8.238	21,578,581	8.666
**Total repeats**		103,467,454	40.31	102,166,277	40.54	76,575,937	32.7	86,137,565	35.91	97,335,863	39.09

D-(sub)genome

		Bdis-Bd1-1		Bhyb-ECID		Bdis-Bd21		BhybABR113D		Bhyb-26D	
**SINE**		524,425	0.188	520,159	0.189	578,602	0.25	561,263	0.208	646,691	0.233
**LINE**		9,778,224	3.514	8,775,509	3.181	9,630,328	3.55	9,670,428	3.591	11,312,808	4.071
	L1	9,549,135	3.432	8,630,798	3.129	9,367,478	3.21	9,437,553	3.504	10,140,262	3.649
	Other	361,989	0.13	178,818	0.07	339,967	0.1	375,370	0.139	1,335,140	0.48
**LTR**		62,378,018	22.416	66,209,381	24.003	60,679,047	22.38	55,714,041	20.687	63,495,031	22.85
	Copia	15,041,865	5.405	18,172,059	6.588	15,749,887	5.81	12,482,402	4.635	17,486,176	6.293
	Gypsy	46,922,353	16.862	47,844,203	17.345	44,798,307	16.52	42,648,093	15.836	45,124,604	16.239
	Other	1,053,543	0.379	7,586,606	2.75	4,096,392	1.51	1,184,103	0.44	1,728,019	0.622
**DNA**		96,403,194	34.643	21,640,727	7.846	22,197,659	8.19	22,053,947	8.189	25,158,957	9.054
	CMC-EnSpm	7,056,952	2.536	6,157,354	2.232	6,344,793	2.34	5,676,006	2.108	7,106,602	2.557
	hAT-Ac	1,192,391	0.428	1,046,434	0.379	1,018,286	0.38	1,186,822	0.441	1,500,334	0.54
	hAT-Tip100	644,571	0.232	673,691	0.244	595,842	0.22	762,703	0.283	1,004,922	0.362
	MuDR	5,322,926	1.913	4,597,099	1.667	5,358,107	1.98	4,974,330	1.847	5,778,241	2.079
	PIF-Harbinger	2,717,781	0.977	2,900,080	1.051	2,724,305	1.01	3,005,884	1.116	3,192,882	1.149
	Other	7,524,670	2.704	6,560,306	2.378	6,929,924	2.56	7,222,802	2.682	7,717,484	2.777
**Transposable elements(TEs) total**		96,403,194	34.643	97,145,776	35.22	93,085,636	34.36	87,999,679	32.675	100,613,487	36.208
**Satellites**		109,020	0.039	159,963	0.058	63,501	0.02	154,530	0.057	49,353	0.018
**Simple_repeats**		8,150,591	2.929	8,693,861	3.152	7,029,862	2.59	6,247,661	2.32	8,561,721	3.081

**Unclassified**	96,403,194	34.643	19,216,348	6.97	16,589,064	6.12	18,389,211	6.828	18,955,117	6.821
**Total repeats**	117,806,652	42.33	117,456,588	42.58	111,673,585	41.18	108,001,607	40.1	120,725,282	43.45

Table S7. Number and total length of rearranged regions between the *B. hybridum* Bhyb-ECIS and Bhyb-ECID subgenomes and its two progenitor species genomes (*B. stacei* Bsta-ECI; *B. distachyon* Bdis-Bd1-1).

**Variation type**	**Count**	**Length**	
		**Bsta-ECI and Bdis-Bd1-1 progenitor species genomes**	**Bhyb-ECID and Bhyb-ECIS subgenomes**
Syntenic regions	6,941	486,636,927	486,582,216
Inversions	147	10,794,675	12,818,815
Translocations	1,539	6,161,398	6,180,190
Duplications (reference)[1]	8,683	20,312,682	-
Duplications (query)	8,444	-	19,153,602
Not aligned (reference)	6,174	88,40,175	-
Not aligned (query)	5,972	-	7,193,580

Table S8. **S**ummary of sequence comparisons between the *B. hybridum* BhS and BhD subgenomes and diploids' genomes at gene level**.** Ks=0 and identical gene number indicated that two homeologous genes have no synonymous substitutions and have the same base sequence, respectively.

B.hybridum subgenome	Diploid genome	No. homeologous gene	No. ks=0 gene	No. identical gene
Bhyb-ECIS	Bsta-ECI	30,206	17,780 (58.86%)	13,372 (44.26%)
Bhyb-ECID	Bdis-Bd1-1	27,171	14,408 (53.02%)	10,314 (37.95%)
Bhyb-ABR113S	Bsta-ABR114	27,873	14,649 (52.55%)	10,550 (37.85%)
Bhyb-ABR113D	Bdis-Bd21	30,371	9,952 (32.7%)	5,744 (18.91%)
Bhyb-26S	Bsta-ABR114	21,633	1,279 (5.91%)	393 (1.81%)
Bhyb-26D	Bdis-Bd21	22,741	1,550 (6.81%)	451 (1.98%)
Bhyb-ABR113S	Bsta-ECI	28,159	12,559 (44.60%)	7,748 (25.51%)
Bhyb-ECIS	Bsta-ABR114	27,352	13,888 (50.77%)	9,585 (35.04%)

Table S9. Summary of reciprocal homoeologous exchange (HE) with replacement regions detected between the two homeologous subgenomes (*B. stacei*-type BhS; *B. distachyon*-type BhD) of **A**) the newly assembled *Brachypodium hybridum* Bhyb-ECI genome and in other 21 resequenced eastern Mediterranean *B. hybridum* allotetraploid genomes, **B**) the *B. hybridum* ABR113 reference genome and in other 5 western Mediterranean *B. hybridum* allotetraploid genomes, **C**) the *B. hybridum* Bhyb-26 reference ancestral genome, **D**) in a total of 30 *B. hybridum* eastern and western Mediterranean and ancestral reference and resequenced genomes.

A) List of HE with replacement (deletions and duplications) identified in 23 eastern Mediterranean *B. hybridum* samples listed by progenitor chromosome positions and corresponding homologous regions of *B. hybridum* ECI genome (Bs01-Bs10: *B. stacei* Bsta-ECI genome; Bd01-Bd05: *B. distachyon* Bdis-Bd1-1 genome).

Deletion (Chromosomal position)	Duplication (Chromosomal position)	No. of samples	Sample description
Bs02: 29140000-29360158<br>BhS2 :0-264859	Bd01: 76050000-76320000<br>BhD1: 74771963-75046193	1	Bhyb-Is-142
Bs09: 21140000-21290000<br>BhS9: 21179549-21337288	Bd05: 1030000-1170000<br>BhD5: 930225-1158973	12	Bhyb-ECI-AS-1,Bhyb-ECI-AS-2,Bhyb-ECI-AS-5,Bhyb-ECI-AS-6,Bhyb-Bal-P1, Bhyb-BdTR6g,Bhyb-IBD107,Bhyb-IBD189, Bhyb-Is-7, Bhyb-Is-151,Bhyb-Is-293, Bhyb-17
Bd01: 72190000-76515858<br>BhD1: 71731963-75245273	Bs02: 25740000-29340000<br>BhS2: 0-3963271	1	Bhyb-Is-146
Bd02: 0-200000<br>BhD2: 0-143506	Bs01: 0-120000<br>BhS1: 31409891-31589311	6	Bhyb-ECI-AS-2,Bhyb-ECI-AS-5,Bhyb-ECI-AS-6, Bhyb-ECI-AS-10, Bhyb-ECI-AS-11, Bhyb-IBD189
Bd04: 9120000-9760000<br>BhD4: 8566149-9198409	Bs10: 190000-370000<br>BhS10: 20547512-20683992	1	Bhyb-Is-142

B) List of HE with replacement (deletions and duplications) identified in 6 western Mediterranean *B. hybridum* samples listed by progenitor chromosome positions and corresponding homologous regions of *B. hybridum* Bhyb-ABR113 genome (Bs01-Bs10: *B. stacei* Bsta-ABR114 genome; Bd01-Bd05: *B. distachyon* Bdis-Bd21 genome).

**Deletion (Chromosomal position)**	**Duplication (Chromosomal position)**	**No. of samples**	**Sample description**
Bd03: 10250000-10710000<br>BhD3: 10449877-10951883	Bs04: 14850000-14960000<br>BhS4: 15318756-15431092	2	Bhyb-ABR113, Bhyb-127
Bs01: 0-200000<br>BhS1: 6427-229678	Bd02: 58890000-59130576<br>BhD2: 59184587-59513736	1	Bhyb-127
Bd01: 48580000-48730000<br>BhD1: 46963323-47121067	Bs07: 9690000-9780000<br>BhS7: 11062662-11111595	1	Bhyb-30
Bs08: 8530000-8690000<br>BhS8: 12338509-12463012	Bd02: 28650000-28810000<br>BhD2: 29233129-29411896	1	Bhyb-30

C) List of HE with replacement (deletions and duplications) identified in 2 D-ancestral (D-anc) *B. hybridum* samples listed by progenitor chromosome positions and corresponding homologous regions of *B. hybridum* Bhyb-26 genome (Bs01-Bs10: *B. stacei* Bsta-ABR114 genome; Bd01-Bd05: *B. distachyon* Bdis-Bd21 genome).

**Deletion (Chromosomal position)**	**Duplication (Chromosomal position)**	**No. of samples**	**Sample description**
Bd04: 5940000-6070000<br>BhD4: 5876414-6395371	Bs10: 5530000-5610000<br>Bh10: 5625963-5883555	1	Bhyb-118-5
Bd04: 30410000-30760000<br>BhD4: 29923795-30226621	Bs10: 11830000-11900000<br>Bh10: 12484358-13821498	1	Bhyb-118-5

D) Summary of potential HE deletion and duplication regions detected in 30 *Brachypodium hybridum* genome samples, which show significant outlier depths. Numbers outside and inside the brackets were obtained from mappings of genomic reads to the merged reference progenitor genomes Ref-East (Bsta-ECI + Bdis-Bd1-1) and Ref-West (Bsta-ABR114 + Bdis-Bd21), respectively. No. HEs: number of HE with replacement swaps; No. Del and No. Dup: number of deletions and duplications; Del-len and Dup-len: deletion and duplication regions' lengths. Origin: evolutionary and geographic origin of the *B. hybridum* samples (D-anc, ancestral western Mediterranean D-plastotype; East-Med: recent eastern Mediterranean S-plastotype; West-Med: recent western Mediterranean S-plastotype).

Sample	No. HEs	No. Del	No. Dup	Del-len	Dup-len	Origin
Bhyb-ABR100	0(0)	149(181)	20(20)	30850250	3790000	Eastern-Med
Bhyb-ABR117	0(0)	148(157)	22(23)	30770250	4090000	Eastern-Med
Bhyb-Adi-P1	0(0)	150(123)	24(21)	37600552	4020000	Eastern-Med
Bhyb-ECI-AS-1	1(1)	128(164)	25(23)	25385354	4490000	Eastern-Med
Bhyb-ECI-AS-10	1(1)	121(137)	22(38)	25070742	4230000	Eastern-Med
Bhyb-ECI-AS-11	1(1)	124(133)	19(33)	26480742	3920000	Eastern-Med
Bhyb-ECI-AS-2	2(1)	118(128)	20(31)	24360742	4260000	Eastern-Med
Bhyb-ECI-AS-5	2(1)	120(136)	23(35)	24620742	4320000	Eastern-Med
Bhyb-ECI-AS-6	2(1)	122(131)	23(35)	24890742	3840000	Eastern-Med
Bhyb-Bal-P1	1(1)	125(158)	22(20)	25229542	4060000	Eastern-Med
Bhyb-BdTR6g	1(0)	128(177)	26(22)	25830000	5900000	Eastern-Med
Bhyb-IBD107	1(0)	123(151)	20(20)	26590552	3570000	Eastern-Med
Bhyb-IBD189	2(1)	131(126)	32(33)	26140742	5000000	Eastern-Med
Bhyb-Is-7	1(1)	132(165)	25(24)	25814784	5900000	Eastern-Med
Bhyb-Is-142	2(1)	136(169)	23(23)	26633742	5280000	Eastern-Med
Bhyb-Is-143	0(1)	125(136)	23(26)	24574594	4940000	Eastern-Med
Bhyb-Is-146	1(1)	130(133)	27(25)	30453774	9210000	Eastern-Med
Bhyb-Is-151	1(1)	131(128)	25(33)	26524784	5740000	Eastern-Med
Bhyb-Is-293	1(2)	132(135)	25(30)	27004784	5760000	Eastern-Med
Bhyb-17	1(0)	139(154)	19(24)	27488004	4600000	Eastern-Med
Bhyb-50	0(0)	127(129)	19(20)	22550137	1790000	Eastern-Med
Bhyb-51	0(0)	119(162)	20(24)	24050552	3460000	Eastern-Med
Bhyb-26	-	366(378)	65(81)	-	-	D-anc
Bhyb-118-5	-	412(412)	64(75)	-	-	D-anc
Bhyb-ABR112	2(0)	167(151)	25(31)	26350137	2364895	Western-Med
Bhyb-ABR113	0(1)	168(161)	13(19)	28430137	997796	Western-Med
Bhyb-30	0(2)	146(146)	22(16)	33791122	2320000	Western-Med
Bhyb-118-8	2(0)	154(129)	23(13)	24820000	850000	Western-Med
Bhyb-123	0(0)	161(154)	23(19)	26140137	1050000	Western-Med
Bhyb-127	2(2)	159(166)	20(17)	29394895	1870576	Western-Med

Table S10. Expression levels of *Brachypodium hybridum* BhD and BhS subgenomic *Ph*1 and *Ph*2 meiotic loci in different plant tissues (leaf, root, spikelet) of ancestral Bhyb-26 and recent Bhyb-ABR113 and Bhyb-ECI *B. hybridum* accessions. Values correspond to TPM of genes. Bhyb-ECI values obtained in this study, Bhyb-ABR113 and Bhyb-26 values obtained in this study through the analysis of TPM data downloaded from Phytozome (https://phytozome-next.jgi.doe.gov/).

	**Leaf**	**Root**	**Spikelet**
***Ph1* locus**			
Bhyb-ECID	0.5016	0.2961	-
Bhyb-ECIS	0.1214	0.5315	-
Bhyb-ABR113D	0.4700	-	1.8761
Bhyb-ABR113S	0.0250	-	1.4200
Bhyb-26D	0.2321	0	-
Bhyb-26S	0.1225	0.0550	-
***Ph2* locus**			
Bhyb-ECID	0	0.6940	-
Bhyb-ECIS	0	0.3946	-
Bhyb-ABR113D	0	-	1.9465
Bhyb-ABR113S	0.0090	-	1.2292
Bhyb-26D	52.511	2.7035	-
Bhyb2-6S	44.134	2.6857	-

Table 11. Summary of Homeologous Expression Bias (HEB) of *Brachypodium hybridum* BhD and BhS genes. **A**) HEB of Bhyb-ECI genes in leaf and root tissues under control (well-watered) and drought conditions. **B**) HEB of constitutionally expressed genes (control conditions) in Bhyb-26. **C**) HEB of constitutionally expressed genes (control conditions) in Bhyb-ABR113. Median calculated from all Log2($TPM_{BhS\text{-}gene}$/$TPM_{BhD\text{-}gene}$) values of BhS-gene and BhD-gene homeologous gene pairs. TPM: transcripts per kilobase per million mapped reads.

A) Bhyb-ECI

**Control**	**Gene number**	**Leaf Percentage (%)**	**Leaf Median**	**Gene number**	**Root Percentage (%)**	**Median**
Filtered homeologous gene pairs	18,830	80.07	0.024	19,920	85.44	0.001
Differentially expressed gene pairs	7,443	31.92	1.034	6,792	29.13	1.004
BhD dominant genes	3,586	15.38	-	3,307	14.18	-
BhS dominant genes	3,857	16.54	-	3,485	14.94	-
All gene pairs	23,313	100.00		23,313	100.00	

**Drought**	**Gene number**	**Leaf Percentage (%)**	**Median**	**Gene number**	**Root Percentage (%)**	**Median**
Filtered homeologous gene pairs	18,907	81.10	0.001	20,314	87.13	0.001
Differentially expressed gene pairs	7,225	30.99	1.004	6,241	26.77	1.025
BhD dominant genes	3,557	15.25	-	3,027	12.98	-
BhS dominant genes	3,668	15.73	-	3,214	13.78	-
All gene pairs	23,313	100.00		23,313	100.00	

B) Bhyb-26

Control	Callus<br>Gene number	Percentage (%)	Median	Floret<br>Gene number	Percentage (%)	Median
Filtered homeologous gene pairs	17,141	83.98	0.015	18,543	90.85	-0.011
Differentially expressed gene pairs	6,305	30.89	1.001	5,279	25.86	-1.014
BhD dominant genes	3,140	15.38	-	2,666	13.06	-
BhS dominant genes	3,164	15.50	-	2,612	12.80	-
All gene pairs	20,411	100		20,411	100	

Control	Leaf<br>Gene number	Percentage (%)	Median	Root<br>Gene number	Percentage (%)	Median
Filtered homeologous gene pairs	17,823	87.32	-0.012	17,988	88.13	-0.023
Differentially expressed gene pairs	5,468	26.79	-1.030	6,075	29.76	-1.035
BhD dominant genes	2,783	13.15	-	3,127	15.32	-
BhS dominant genes	2,684	13.63	-	2,947	14.44	-
All gene pairs	20,411	100		20,411	100	

C) Bhyb-ABR113

Control	Leaf			Spike		
	**Gene number**	**Percentage (%)**	**Median**	**Gene number**	**Percentage (%)**	**Median**
Filtered homeologous gene pairs	17,857	78.00	-0.004	20,790	90.81	-0.03
Differentially expressed gene pairs	6,757	29.51	1.002	5,139	22.45	-1.004
BhD dominant genes	3,304	14.43	-	2,558	11.17	-
BhS dominant genes	3,452	15.08	-	2,580	11.27	-
All gene pairs	22,892	100		22,892	100	

Table S12. Summary of the number of biased expressions of dominant *Brachypodium hybridum* genes from homeologous BhD and BhS gene pairs in Bhyb-ECI, Bhyb-ABR113 and Bhyb-26.

	BhD dominant	BhS dominant	Total
Bhyb-ECI	673	745	1418
Bhyb-ABR113	1115	1156	2271
Bhyb-26	635	634	1269

Table S13. Number of biased expressions of dominant *Brachypodium hybridum* Bhyb-ECI genes from homeologous BhD and BhS gene pairs in different tissues (leaf, root) and different conditions (well-watered, drought).

Class	Dominant subgenome	leaf		Root	
		Count	Percentage (%)	Count	Percentage (%)
Only biased in well-watered condition	BhS	1,217	12.66	1,462	15.94
	BhD	1,170	12.17	1,467	16.00
Only biased in drought condition	BhS	1050	10.92	1,171	12.77
	BhD	1,119	11.64	1,207	13.16
biased in both well-watered and drought conditions	BhS	2,548	26.51	1,878	20.48
	BhD	2,346	24.41	1,675	18.27
	BhD changed to BhS	70	0.73	165	1.80
	BhS changed to BhD	92	0.96	145	1.58

Supplementary Figures: *Brachypodium hybridum*

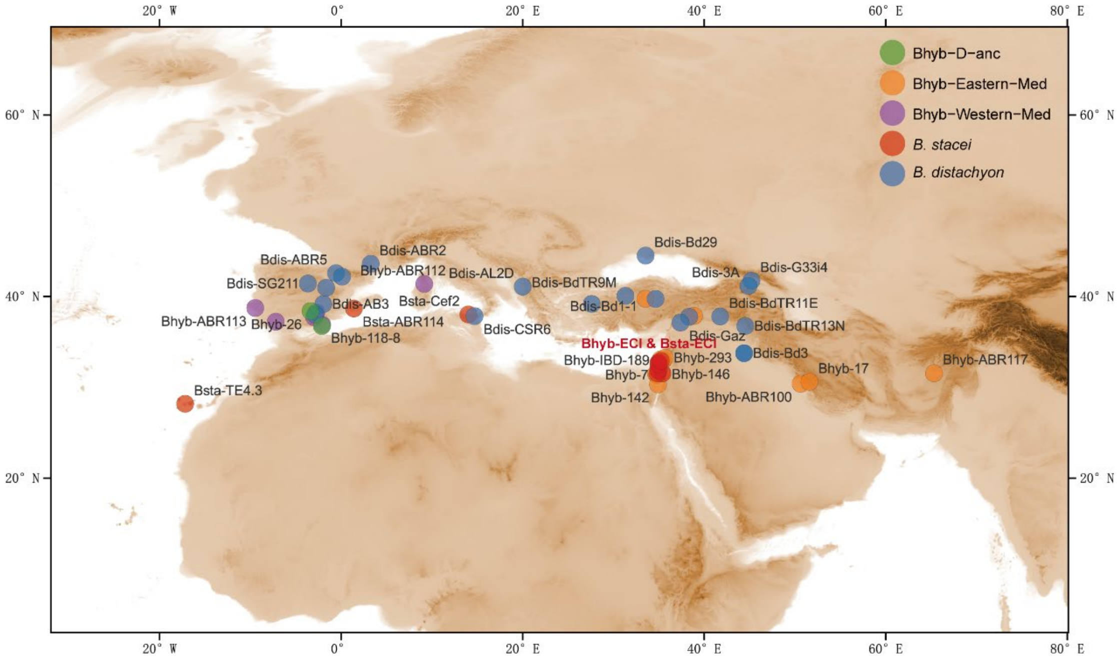

Figure S1. Map showing the geographic distribution of the studied *Brachypodium hybridum* accessions in its native circum-Mediterranean region (ancestral (Bhyb-D-anc): green dot; recent West Mediterranean (Bhyb-Western-Med): purple dot; recent East Mediterranean (Bhyb-Eastern-Med): orange dot) and the progenitor species *B. stacei* (red dots) and *B. distachyon* (blue dots) accessions used in the comparative genomic analyses.

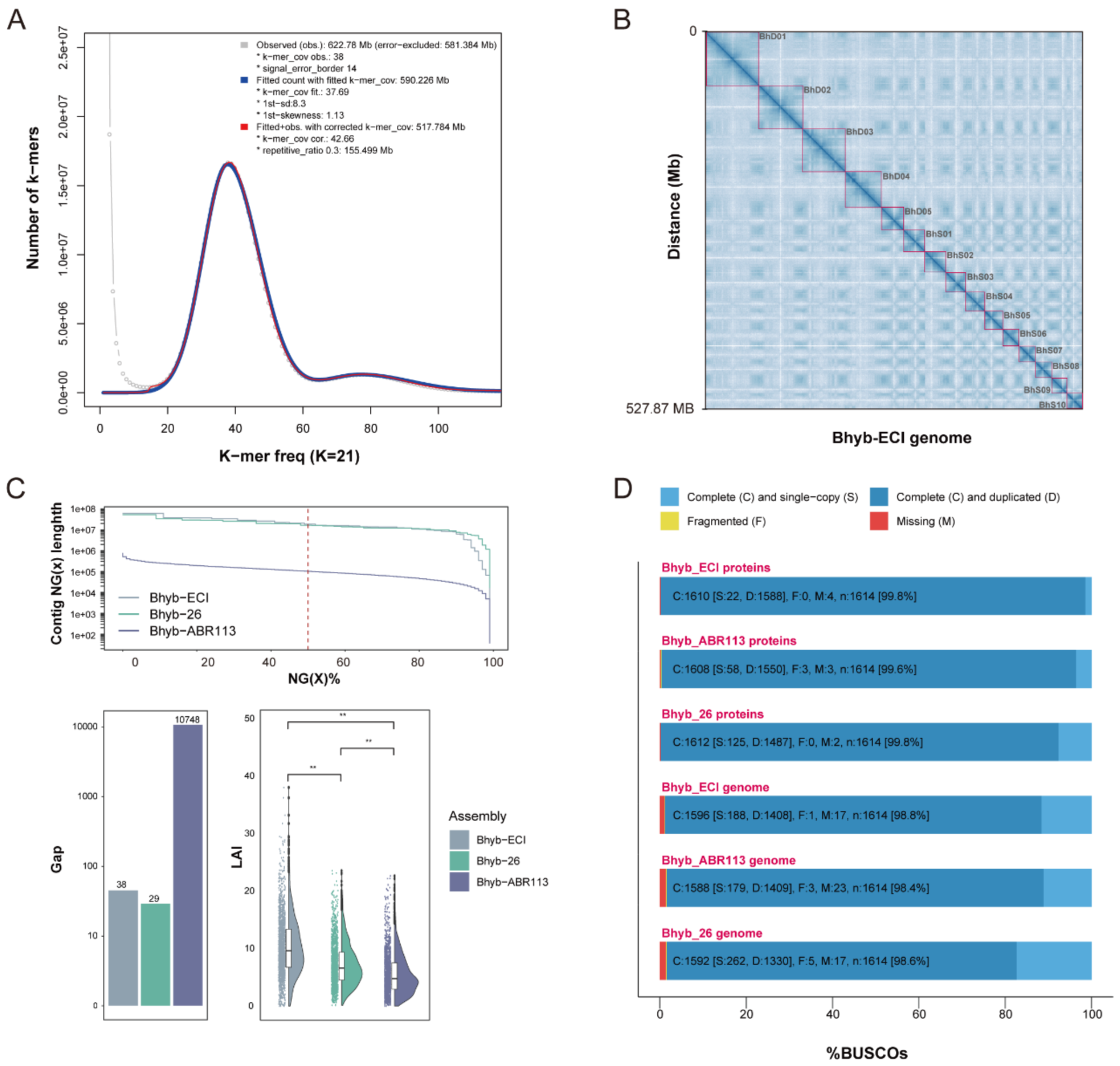

Figure S2. Genome survey of the newly sequenced *B. hybridum* Bhyb-ECI reference genome from Evolution Canyon I (see Supplementary table S2 for additional information)**. A)** K-mer frequency. K-mer size was set at 21 and the default parameters were set with FindGSE. **B)** Heat maps showing densities of Hi-C interactions between contigs, with dark blue indicating a high density of interactions and distinct chromosomes marked by red boxes. BhD01-BhD05, *B. distachyon*-type chromosomes; BhS01-BhS10, *B. stacei*-type chromosomes. **C)** Overview of assembly NG values, gap numbers and the LTR Assembly Index (LAI) for the Bhyb-ECI, Bhyb-26, and Bhyb-ABR113 genomes (colour codes indicated in the charts). Calculated NG contig lengths range from 1 to 100% and the contig length (in bp) for particular thresholds are shown on the y-axis (plotted on a log scale). The dashed vertical line indicates the NG50 contig length. **p-value < 0.01 (Wilcoxon test). **D)** BUSCO gene assessments of the Bhyb-ECI genome, the Bhyb-26 genome, and the reference genome Bhyb-ABR113 v1.1 (Phytozome https://phytozome-next.jgi.doe.gov). Color codes for gene types are indicated in the chart. genome: run BUSCO in "genome" mode; protein: run BUSCO in "proteins" mode.

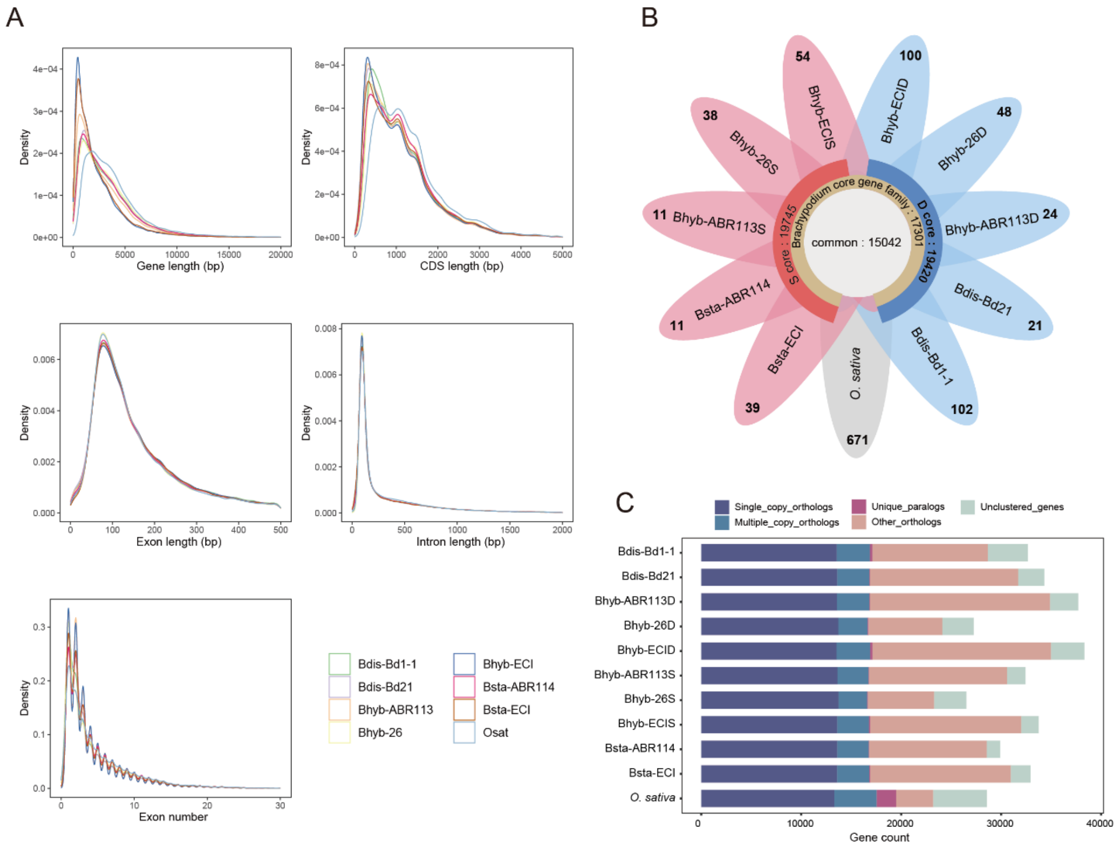

Figure S3. Comparison of gene structure characteristics and gene family in the newly sequenced *B. hybridum* Bhyb-ECI reference genome from Evolution Canyon I (Israel) to those in other assemblies (reference genomes of *B. stacei* Bsta-ECI, Bsta-ABR114, *B. distachyon* Bdis-Bd21, Bdis-Bd1-1, *B. hybridum* Bhyb-ABR113, Bhyb-26, and *Oryza sativa*.) (Gordon et al. 2020; Mu et al. 2023, Scarlett et al 2022). **A)** gene length; CDS length; exon length; intron length; exon number. **B)** Pan-genomic comparison of the number of gene families in different levels, the numbers in petal indicated amount of unique families of each (sub-)genome, the circle in inner showed core gene families number in different comparison levels. **C)** Classification of genes into large gene types in *Brachypodium* and *O. sativa*. Color codes for genomes and for gene types are indicated in the respective charts.

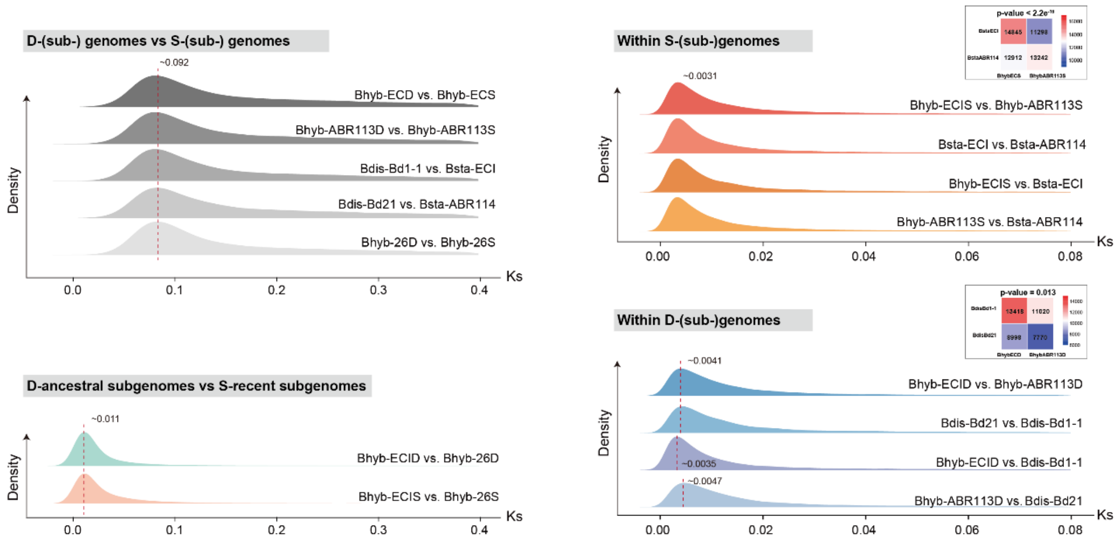

Figure S4. Inter- and intra-(sub-)genome comparisons of ks density using orthologous nuclear gene pairs between compared S and D genomes and subgenomes. The heatmap shows the number of gene pair that had no synonymous substitutions (Ks = 0, excluded in ks density analysis) between the pairwise compared genomes/subgenomes (chisq.test).

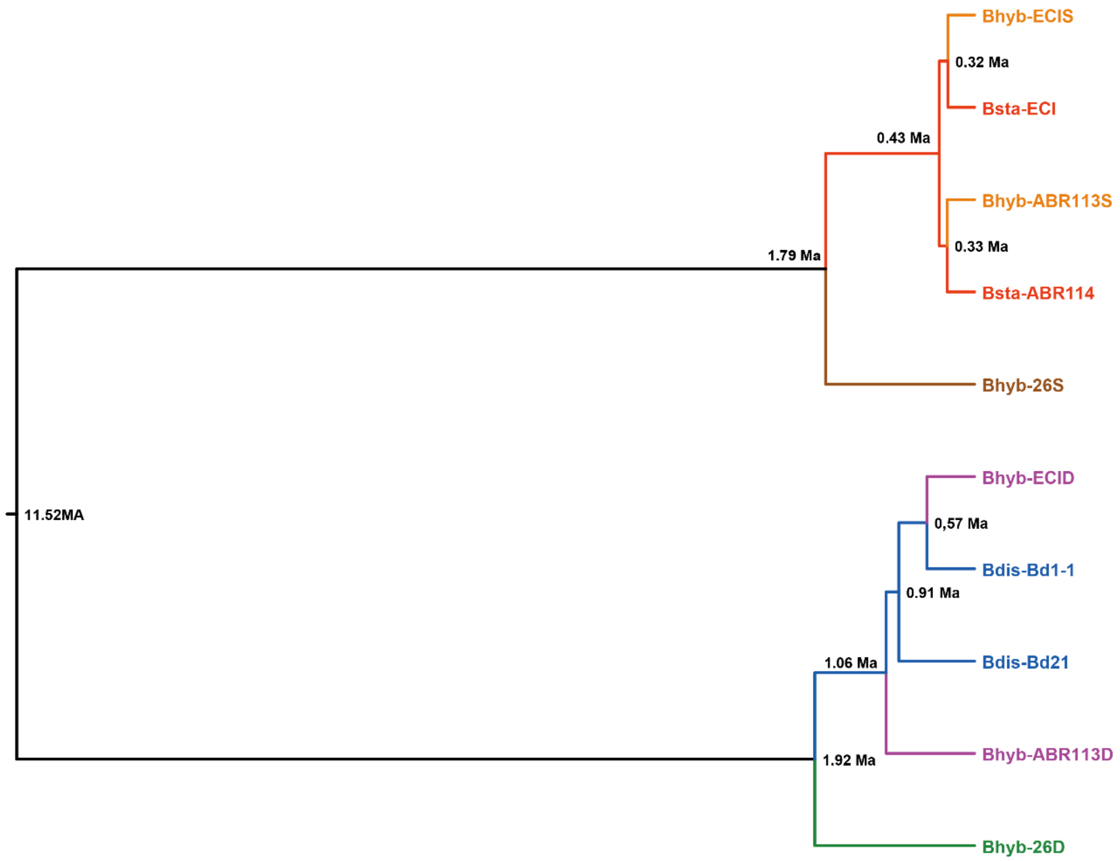

Figure S5. Coalescent-based dated SNAPP tree of *B. stacei* (Bsta-ABR114, Bsta-ECI) and *B. distachyon* (Bdis-Bd21, Bdis-Bd1-1) progenitor genomes and of *B. hybridum* (D-an Bhyb26, West-Med Bhyb-ABR113, East-Med Bhyb-ECI) BhS and BhD subgenomes based on 30,000 syntenically aligned SNPs retrieved from 12,481 nuclear single-copy orthologous genes (SCOGs) showing estimated divergence times for branches. Cross-bracing estimations were obtained separately for each allopolyploidization event using the closest available progenitor genomes for each allotetraploid (Bsta-ECI and Bdis-Bd1-1 for East-Med Bhyb-ECI, Bsta-ABR114 and Bdis-Bd21 for West-Med Bhyb-ABR113, and Bsta-ABR114 and Bdis-Bd21 for the ancestral D-an Bhyb26) (see text and fig. 2B).

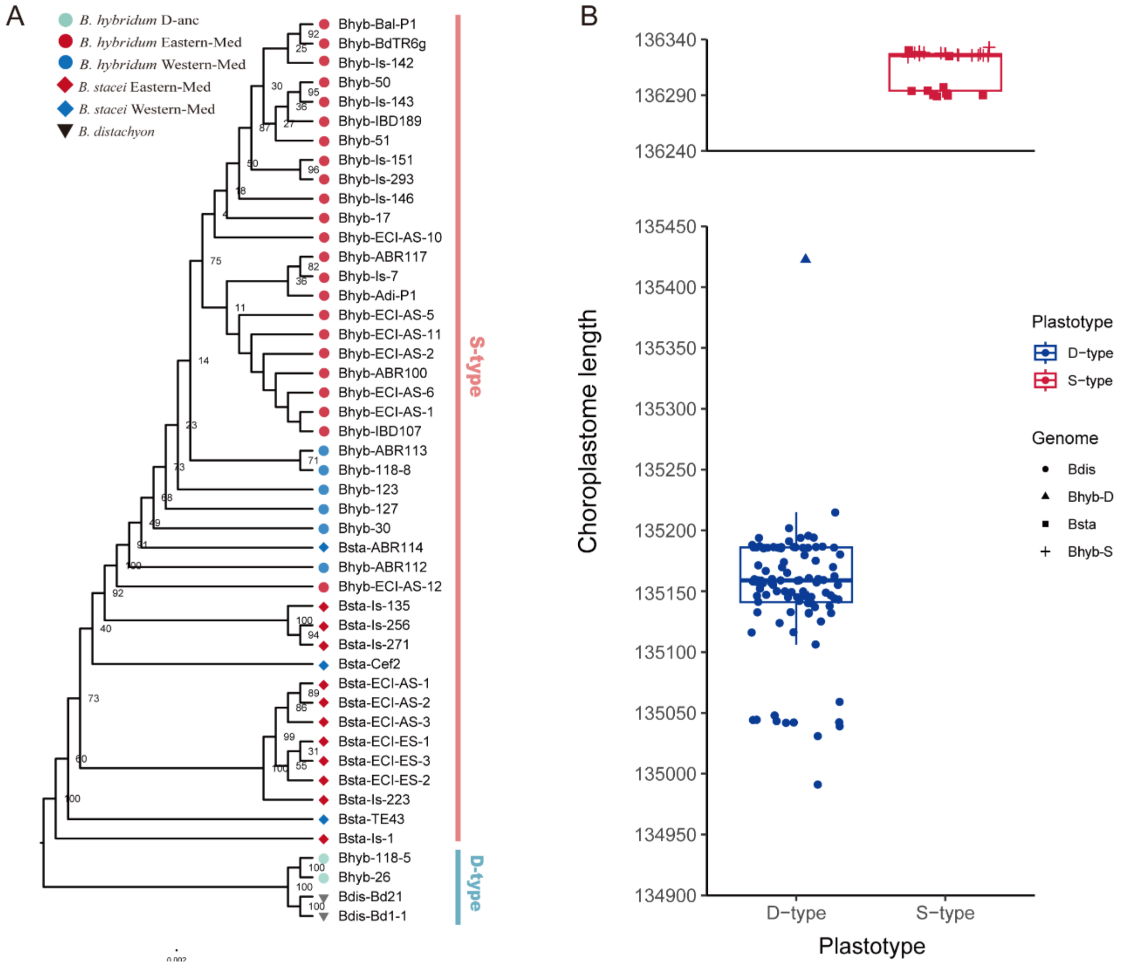

Figure S6. Phylogenomic tree based on whole plastome sequences of *Brachypodium hybridum, B. stacei* and *B. distachyon* accessions and plastome lengths summary of newly assembled plastomes and other published plastomes. **A)** Maximum-likelihood tree of *B. hybridum* individuals from ECI plus available plastomes of other eastern, central and western Mediterranean *B. hybridum* and progenitor species *B. distachyon* and *B. stacei* accessions. Boostrap support values are indicated on branches. **B)** Plastome lengths of the reference genomes and other accessions of the three annual *Brachypodium* species; different plastotypes and species are marked by color and shape (see Sancho et al. (2018) and Gordon et al. (2020).

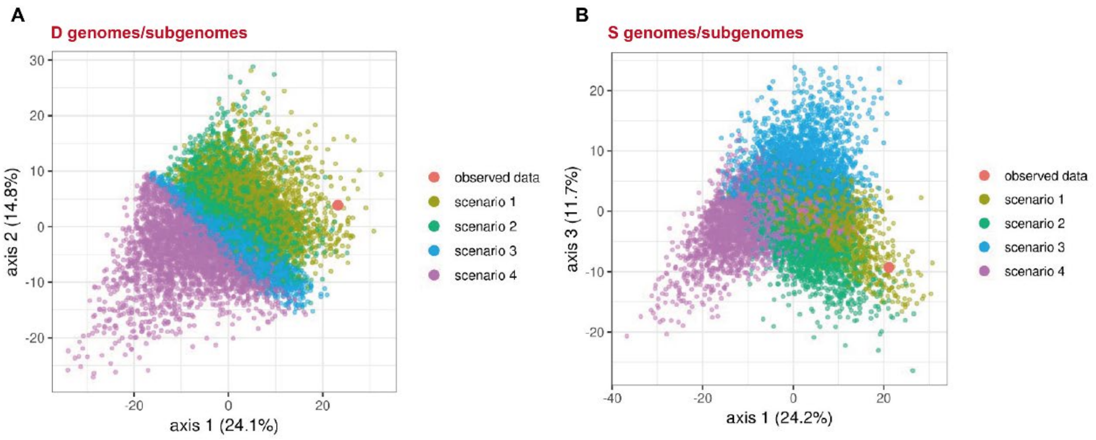

Figure S7. Bidimensional PCA plots of observed data and of simulated data for each of the four tested evolutionary scenarios on the alternative origins of *Brachypodium hybridum* allotetraploids (see fig. 2C and supplementary table S5). **A**) D genomes/subgenomes. **B**) S genomes/subgenomes. 4,000 simulated data sets per scenario were generated with DIYABC-RF. The observed data fell within the clouds of simulated data.

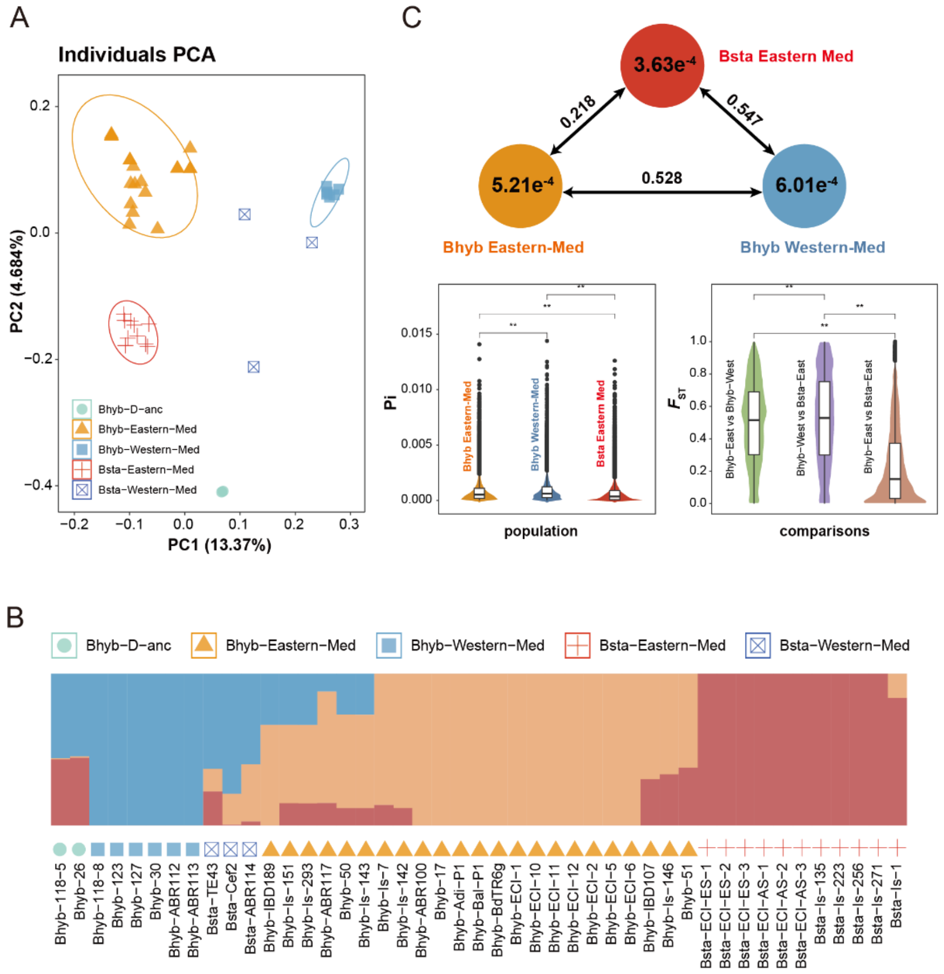

Figure S8. Genome structure analysis based on nuclear syntenic SNP data of *B. hybridum* (BhS subgenome) and its progenitor species *B. stacei*. **A**) Bidimensional Principal Component Analysis (PCA) plots of nuclear syntenic SNPs of S-genomes discriminating the *B. hybridum* S-plastotype plus *B. stacei* eastern vs western Mediterranean populations along PCA1 (10.1% accumulation of variance) and the *B. hybridum* D-plastotype along PCA2 (4.32%). **B**) Population structure analysis plot of S-genomes for the best (K=3) genomic groups, showing differentiating the eastern *B. hybridum* populations group, the eastern *B. stacei* populations group, and western *B. stacei* + *B. hybridum* populations group. **C**) The nucleotide diversity (π) and pair-wised population divergence (F_{ST}) were examined across the three main populations groups. The value within each circle represents the calculated π for that particular group, while the value along each line indicates the F_{ST} between two adjacent groups. The distribution of π and F_{ST} values are also shown in subplots. **p-value < 0.01 (Wilcoxon test).

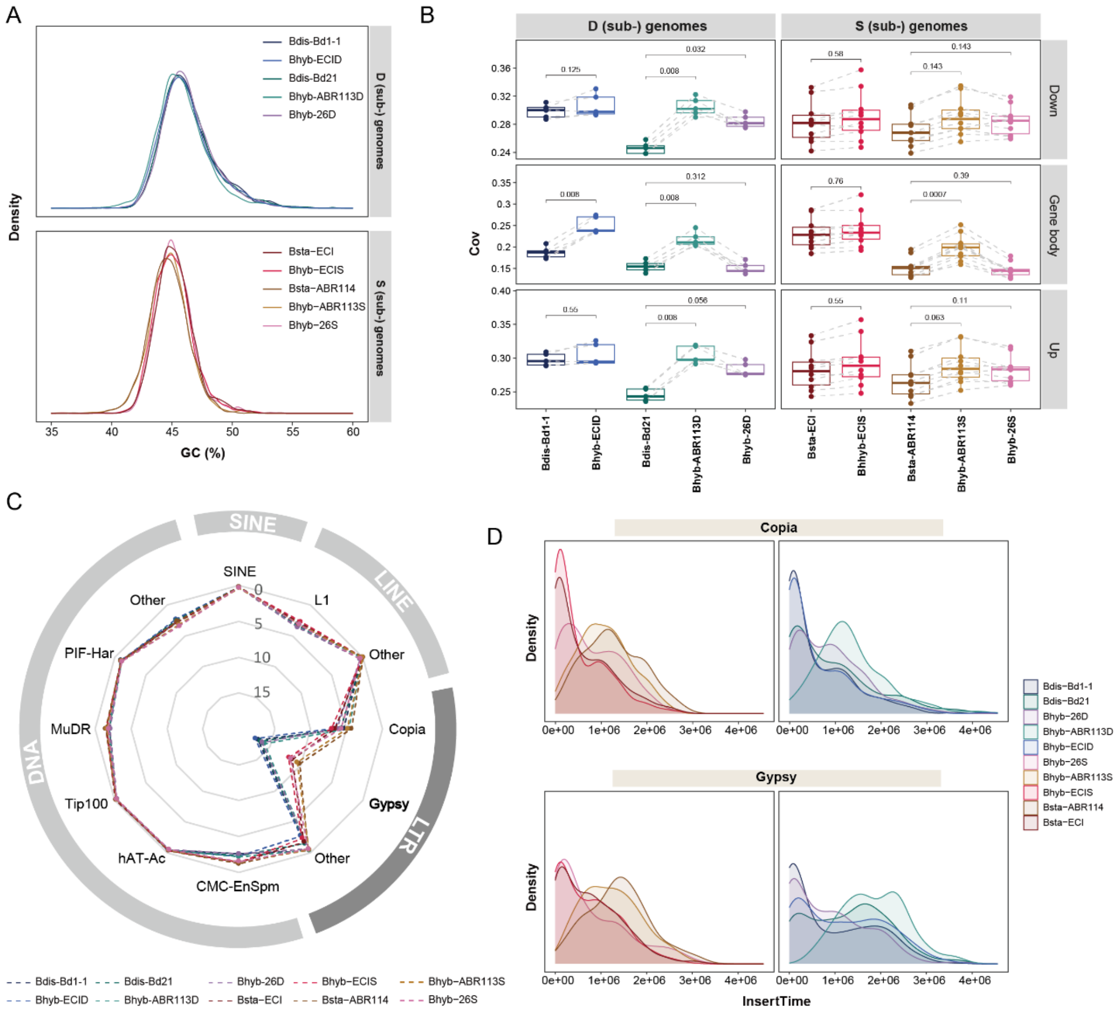

Figure S9. Comparisons of genomic features between the *Brachypodium hybridum* Bhyb-ECID and Bhyb-ECIS subgenomes and those of other allotetraploids (Bhyb-ABR113, Bhyb-26) and the genomes of the progenitor species *B. distachyon* (Bdis-Bd1-1, BdisBd21) and *B. stacei* (Bsta-ECI, Bsta-ABR114). **A)** GC content. **B)** Repetitive content coverage nearby gene regions; down indicates gene downstream 0-10k sequence, up gene upstream 0-10k sequence, and gene the encoding region containing CDSs and introns. The dashed lines link homologous chromosome pairs between the subgenomes and the respective progenitor genomes. **C)** Composition of repetitive sequences per main repeat categories. The coordinates represent the ratio of each category to the total length of the corresponding genome or subgenome. **D)** Estimated insertion times of Copia and Gypsy retrotransposons in the *B. distachyon* and *B. stacei* genomes and BhD and BhS subgenomes of *B. hybridum*. Color and symbol codes for *B. distachyon* (BdisBd1-1, BdisBd21) and *B. stacei* (Bsta-ECI, Bsta-ABR114) genomes, and *B. hybridum* (Bhyb-ECI, Bhyb-ABR113, Bhyb-26) BhD and BhS subgenomes are indicated in the corresponding charts of each subfigure.

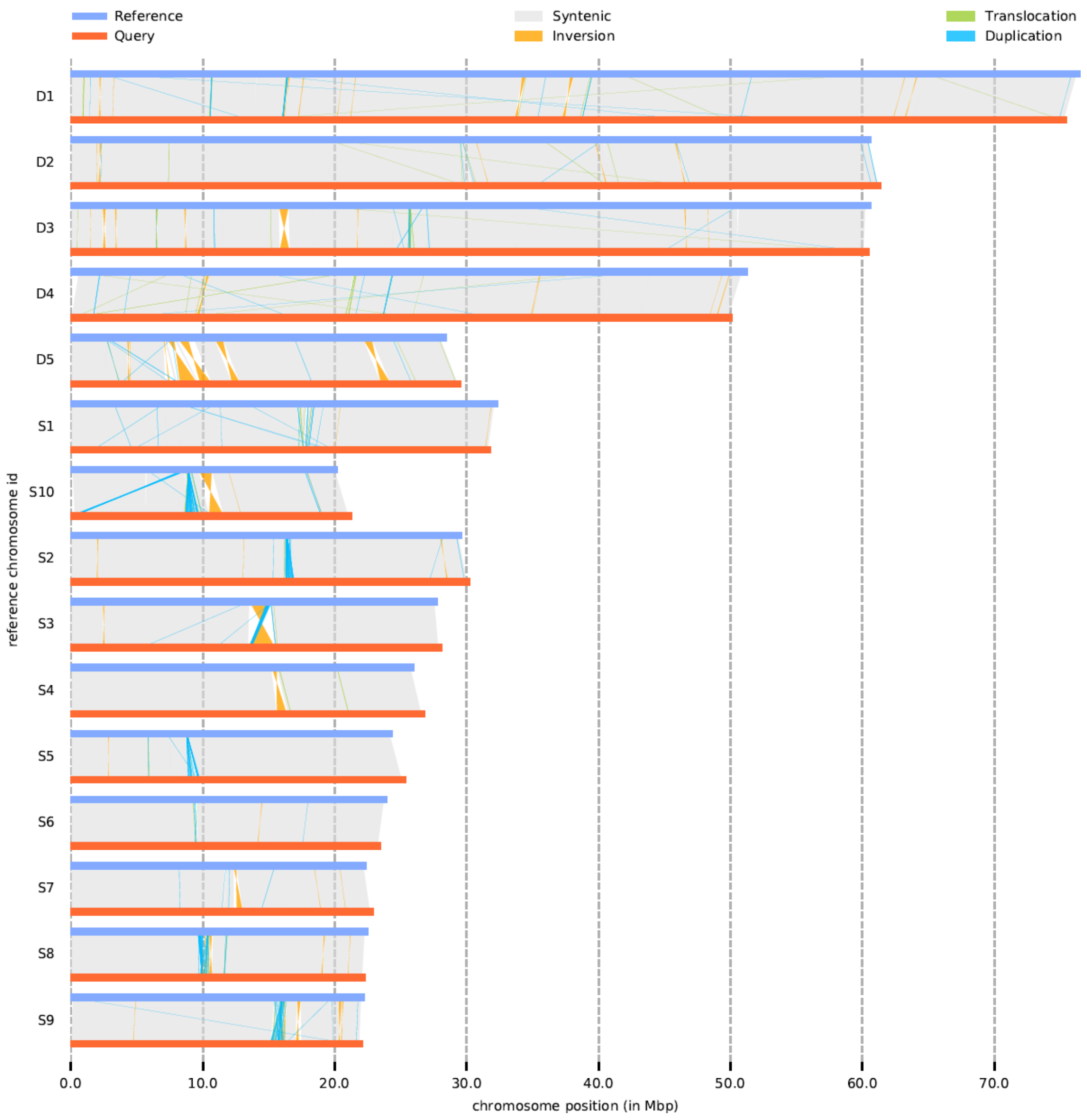

Figure S10. The landscape of genomic rearrangements between the *B. hybridum* Bhyb-ECID and Bhyb-ECIS subgenomes (query) and its two progenitor species genomes (*B. stacei* Bsta-ECI and *B. distachyon* Bdis-Bd1-1). D1-D5: *B. distachyon* and BhD chromosomes, S1-S10: *B. stacei* and BhS chromosomes. Color codes for different rearrangement are indicated in the chart.

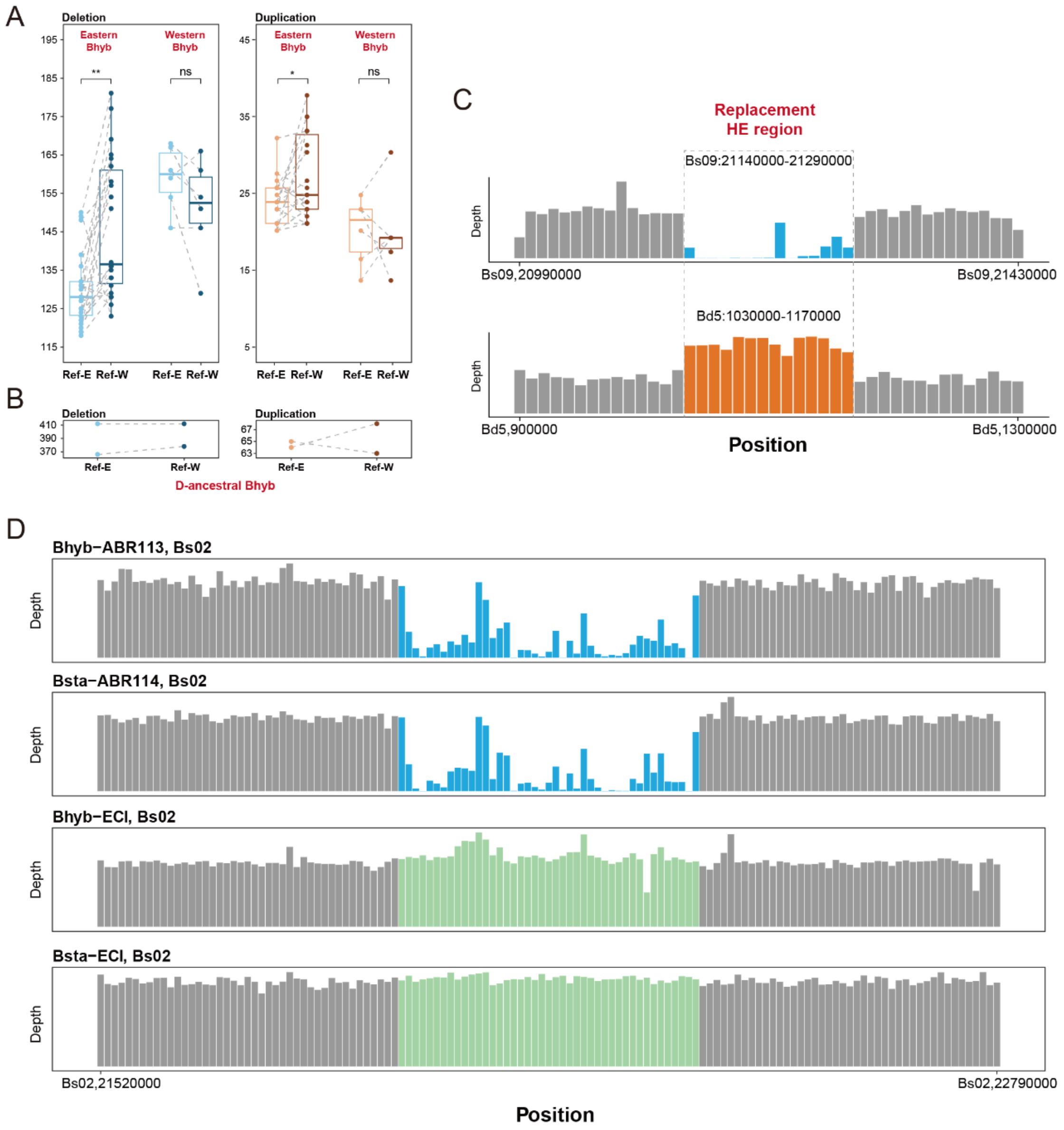

Figure S11. Homeologous exchange (HE) events detected in the newly assembled *B. hybridum* Bhyb-ECI genome and in other 29 *B. hybridum* allotetraploid genomes. Deletion and duplication tests resulting from comparisons of mapped *B. hybridum* genome reads to different combined reference progenitor genomes (Ref-East: concatenated Bsta-ECI + Bdis-Bd1-1; Ref-West: concatenated Bsta-ABR114 + Bdis-Bd21). **A)** Recent Eastern (Bhyb-ECI) and Western Mediterranean (Bhyb-ABR113) samples. **B)** D-ancestral (Bhyb-26) sample. **p-value < 0.01, *p-value < 0.05 (paired Wilcoxon-test). **C)** Example of *B. hybridum* Bhyb-ECI HEs in homeologous chromosomes BhS09-BhD05 detected when mapping Bhyb-ECI reads to the merged diploid progenitor species genomes (Bsta-ECI + Bdis-Bd1-1) showing a deletion (decreasing depth) and its reciprocal duplication (increasing depth) in regions homologous to those of the progenitor genomes, Bs09:21140000-21290000 and Bd5:1120000-1260000, respectively (see also supplementary table S9A). **D)** Different HE patterns between western Mediterranean Bhyb-ABR113 and eastern Mediterranean Bhyb-ECI *B. hybridum* BhS subgenomes and their respective *B. stacei* progenitor genomes Bsta-ABR114 and Bsta-ECI: deletion in chromosomal region Bs02 21520000-22790000（Bsta-

ECI genome）in Bhyb-ABR113S and Bsta-ABR114; absence of deletion in Bhyb-ECI and Bsta-ECI.

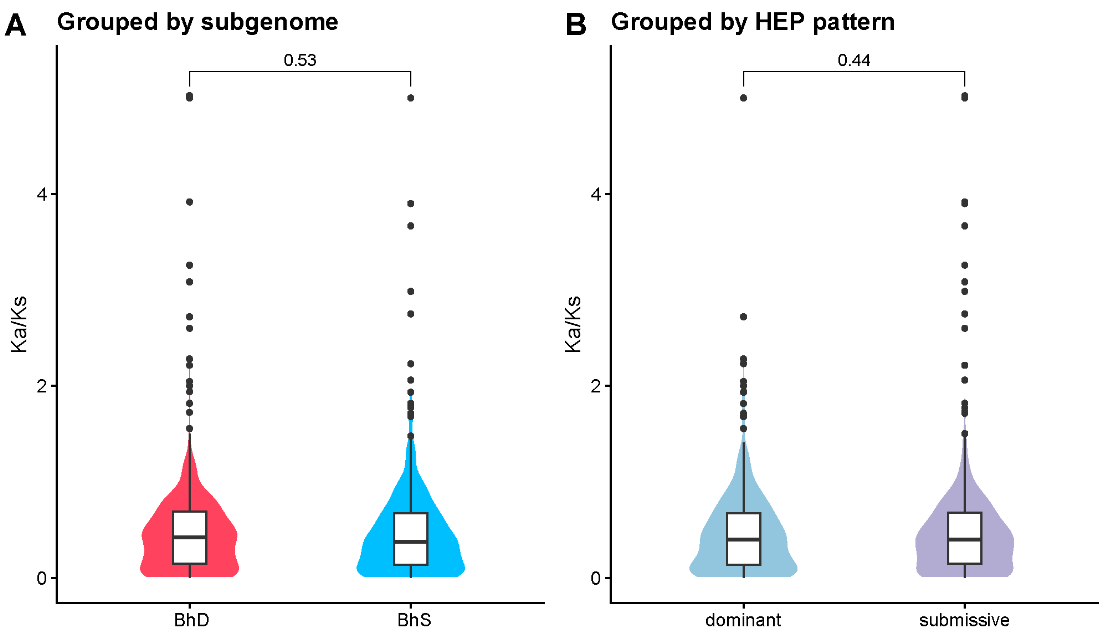

Figure S12. Comparisons of Ka/Ks distribution of orthologous/homeologous gene pairs between the Bhyb-ECID and Bhyb-ECIS subgenomes and its two progenitor species' genomes (*B. distachyon* D (Bdis-Bd1-1); *B. stacei* S (Bsta-ECI) (t-test). **A)** Grouped by genome/subgenome (BhD-type, BhS-type). **B)** Grouped by expression bias type (dominant, submissive). (Wilcox paired test).

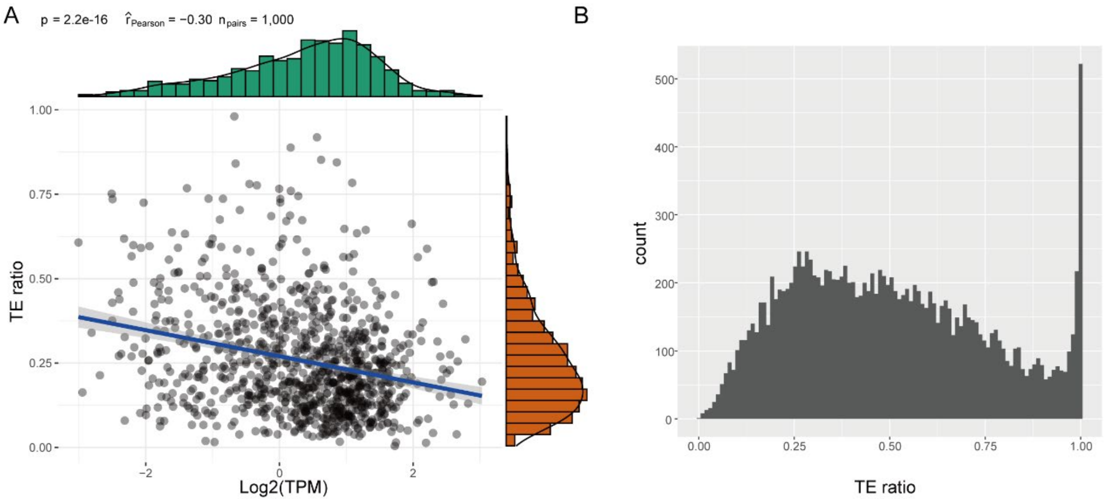

Figure S13. The relationship between gene expression level in transcripts per million (TPM) and transposable element (TE) ratio nearby genes of the Bhyb-ECI genome from Evolution Canyon I. TE ratio of nearby genes was calculated in 2kb upstream to 2kb downstream windows of the gene. **A)** Gene expression is negatively correlated with TE density near the genes in Bhyb-ECI, 1000 genes were randomly selected to be visualized. **B)** Histogram plot of TE ratio in non-expressed genes (TPM = 0). Comparisons of TE density in genes show stability in all case study groups.

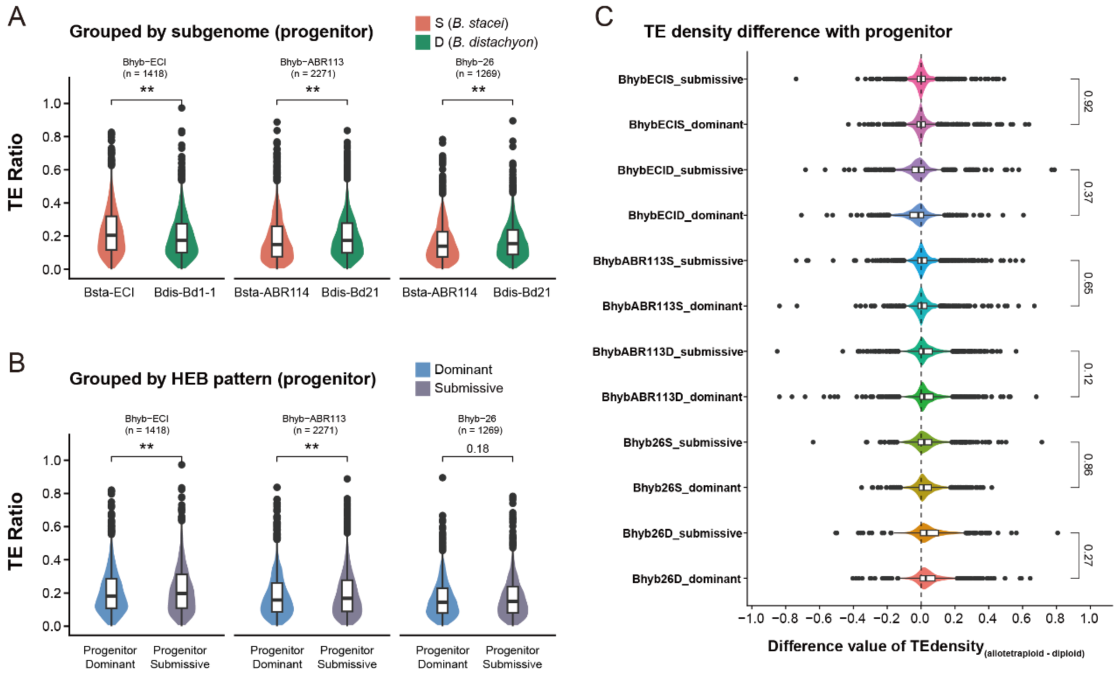

Figure S14. Comparison of TE density near the orthologous gene of stable biased homologous gene pairs in all case study groups, Bhyb-ECI (progenitor genomes: Bsta-ECI and Bdis-Bd1-1), Bhyb-ABR113 (progenitor genomes: Bsta-ABR114 and Bdis-Bd21) and Bhyb-26 (progenitor genomes: Bsta-ABR114 and Bdis-Bd21). **A)** Grouped by subgenome (BhD, BhS) and **B)** grouped by expression bias type (HEB; Dom: dominant, Sub: submissive). ** p-value < 0.01 (paired Wilcoxon test).**C)** The distribution of TE density near the gene between progenitor genomes and the corresponding allotetraploid's D and S subgenomes. p-value calculated by Wilcoxon test.

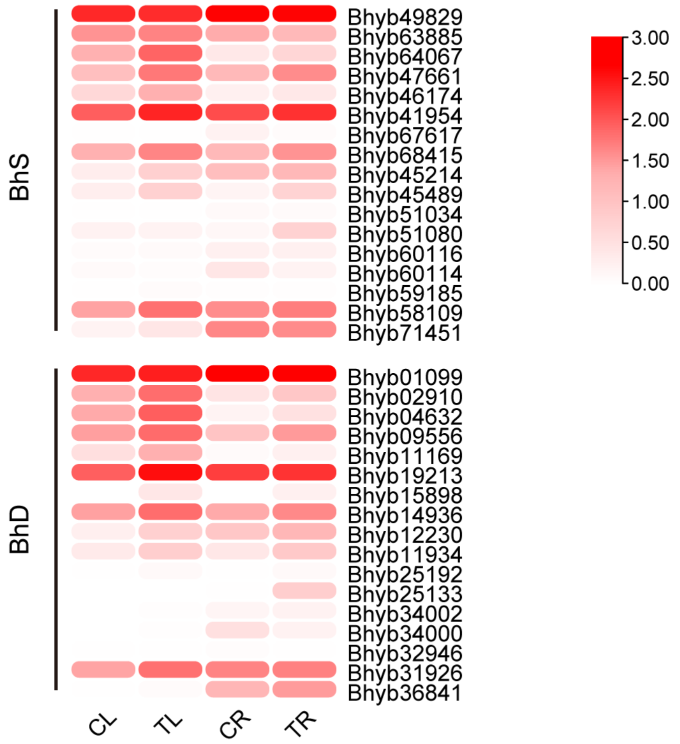

Figure S15. Heatmap of differential gene expression levels of stress-related NAC genes in the *Brachypodium hybridum* Bhyb-ECI BhD and BhS subgenomes. L, leaf tissue, R, root tissue, C control (well-watered) conditions, T treatment (drought) conditions. The chart legend is Log_{10}(TPM).
